# Hematopoietic plasticity mapped in *Drosophila* and other insects

**DOI:** 10.7554/eLife.78906

**Published:** 2022-08-03

**Authors:** Dan Hultmark, István Andó

**Affiliations:** 1 Department of Molecular Biology, Umeå University Umeå Sweden; 2 Biological Research Centre, Institute of Genetics, Innate Immunity Group, Eötvös Loránd Research Network Szeged Hungary; École Polytechnique Fédérale de Lausanne Switzerland; University of California, Los Angeles United States

**Keywords:** *Drosophila*, hemocytes, immunity, hematopoiesis, mosquitoes, lepidoptera

## Abstract

Hemocytes, similar to vertebrate blood cells, play important roles in insect development and immunity, but it is not well understood how they perform their tasks. New technology, in particular single-cell transcriptomic analysis in combination with *Drosophila* genetics, may now change this picture. This review aims to make sense of recently published data, focusing on *Drosophila melanogaster* and comparing to data from other drosophilids, the malaria mosquito, *Anopheles gambiae*, and the silkworm, *Bombyx mori*. Basically, the new data support the presence of a few major classes of hemocytes: (1) a highly heterogenous and plastic class of professional phagocytes with many functions, called plasmatocytes in *Drosophila* and granular cells in other insects. (2) A conserved class of cells that control melanin deposition around parasites and wounds, called crystal cells in *D. melanogaster*, and oenocytoids in other insects. (3) A new class of cells, the primocytes, so far only identified in *D. melanogaster*. They are related to cells of the so-called posterior signaling center of the larval hematopoietic organ, which controls the hematopoiesis of other hemocytes. (4) Different kinds of specialized cells, like the lamellocytes in *D. melanogaster*, for the encapsulation of parasites. These cells undergo rapid evolution, and the homology relationships between such cells in different insects are uncertain. Lists of genes expressed in the different hemocyte classes now provide a solid ground for further investigation of function.

## Introduction

Like most other animals, the fruit fly *Drosophila melanogaster* has blood cells patrolling all parts of the organism (Rizki, 1978; Rizki, 1984; Meister and Lagueux, 2003). These cells, the hemocytes, attack pathogens, participate in blood clotting and wound healing, and mediate the remodeling of tissues during development. Our understanding of how hemocytes carry out these tasks is surprisingly limited, at least on the molecular level. This may seem surprising, considering the popularity of this model organism. However, important steps forward have been taken during the past two decades. Antibodies and genetic markers have been developed to follow the hemocytes in vivo and manipulate their activities (Evans et al., 2014), and the hematopoietic events that control the production of these cells have now been characterized in considerable detail, as summarized in excellent reviews (Evans et al., 2003; Martinez-Agosto et al., 2007; Honti et al., 2014; Ramond et al., 2015; Gold and Brückner, 2015; Letourneau et al., 2016; Csordás et al., 2021). Our present knowledge about the role of hemocytes in immunity has been reviewed by Carton et al., 2008; Williams, 2007; Theopold and Dushay, 2007; Wang et al., 2014; and Yang et al., 2020, and their involvement in embryology and wound healing by Fauvarque and Williams, 2011; Evans and Wood, 2014; Theopold et al., 2014; and Ratheesh et al., 2015. For a recent comprehensive review of the entire field, with an emphasis on hematopoiesis, see Banerjee et al., 2019.

In spite of this progress, many central questions remain to be answered, but the development of single-cell sequencing technology may change the picture. During the past years, this technique has generated a wealth of data that may look incoherent, but that could potentially change the field. This review is an attempt to summarize and critically analyze this new information in the light of what we knew before.

## What we knew before (briefly)

### Hemocyte classes

Working with larvae of *D. melanogaster*, Rizki, 1957 identified three morphologically defined classes of hemocytes, which he called plasmatocytes, crystal cells, and lamellocytes. He also described a fourth class, the podocytes, which he regarded as intermediates between plasmatocytes and lamellocytes. The *plasmatocytes* are relatively small hemocytes with a slightly granular cytoplasm. They constitute the majority of hemocytes and participate in the immune defense by phagocytizing bacteria and other foreign objects. They also attach to larger parasites, initiating their encapsulation. Plasmatocytes also participate in the reshaping of tissues during embryonic development and metamorphosis and play an active role in wound healing. *Crystal cells* are distinguished by large crystal-like inclusions that contain components of the phenoloxidase system, primarily phenoloxidase itself. Phenoloxidase is the key enzyme that generates melanin, a black pigment that is deposited in capsules around parasites and in wounds. When disturbed, crystal cells disintegrate and release their contents (Rizki and Rizki, 1959; Bidla et al., 2007). *Lamellocytes* are typically absent in healthy larvae, but they are produced in large numbers in response to infections by parasitoid wasps. Wounding induces a similar, but usually weaker, response. Some labs also report substantial spontaneous production of lamellocytes in uninfected larvae, during a brief period at the early wandering stage (Rizki, 1957; Shrestha and Gateff, 1982; Leitão et al., 2020). The lamellocytes are major constituents of the capsules that are formed around parasites. Finally, the *podocytes* were described to be similar to the plasmatocytes, differing from the latter only by their long cytoplasmic filaments. Rizki’s observations were later corroborated by several other workers (Nappi and Streams, 1969; Shrestha and Gateff, 1982), but the relationship between the different classes and the status of the podocytes has been questioned. Some observations indicate that the podocytes undergo endoreplication, unlike the mitotically active plasmatocytes (Stewart and Denell, 1993).

In healthy wild-type third-instar larvae, Rizki, 1957 estimated that plasmatocytes constitute about 90–95% of all circulating hemocytes, while the remaining 5–10% are crystal cells. Although highly variable, these proportions were later confirmed by others (Nappi and Streams, 1969; Shrestha, 1979; Shrestha and Gateff, 1982). Similarly, using molecular markers, Leitão and Sucena, 2015 found 4–17% crystal cells in the sessile population. Others find fewer crystal cells, only 2–5% (Lanot et al., 2001; Brehélin, 1982; Meister and Lagueux, 2003). The discrepancies could be due to genetic differences or perhaps to the difficulties to accurately count the crystal cells, which tend to disintegrate when they are disturbed. Crystal cells are depleted in wasp-infested larvae (Nappi and Streams, 1969).

While lamellocytes have only been observed in larvae, plasmatocytes and crystal cells are also found in embryos and adults (Tepass et al., 1994; Kurucz et al., 2007b; Ghosh et al., 2015). Pupae have plenty of active plasmatocytes but essentially no crystal cells (Grigorian et al., 2011). A fair proportion of the larval hemocytes (about 50%) are found circulating in the hemocoel, while the remainder stay attached to the extracellular matrix in a segmental pattern under the skin and on internal organs. There is exchange between the sessile and circulating populations, and the sessile cells can rapidly be mobilized when the animal is parasitized or wounded. In the embryo, the hemocytes are not circulating freely, but they are motile and can actively migrate to the sites where they are needed (Fauvarque and Williams, 2011; Evans and Wood, 2014; Ratheesh et al., 2015). By contrast, sessile hemocytes in the larvae are immobile, but they regain the capacity to migrate in the prepupal stage (Sampson and Williams, 2012), where they play a very active role as phagocytes in the turnover of metamorphosing tissues (Ghosh et al., 2020).

Early workers in the field observed a substantial spontaneous production of lamellocytes in healthy late third-instar larvae (Rizki, 1957; Shrestha and Gateff, 1982), while others (like ourselves) rarely see any. This difference may have a genetic, or possibly epigenetic, explanation. Leitão et al., 2020 found that the larvae in an outbred *Drosophila* population, established from wild-caught females, were constitutively producing lamellocytes, and that this trait could be affected by selection. The proportion of constitutively produced lamellocytes was considerably increased in a population that had been raised under intense parasitoid wasp parasitism, and these larvae were also more resistant to parasitism. Standard lab stocks, which have been bred for decades without such selective pressure, may have lost the constitutive lamellocyte phenotype.

### Embryonic and adult hemocytes

*Drosophila* hemocytes have been most thoroughly investigated in third-instar larvae, from which large numbers of hemocytes are conveniently available. Embryonic hemocytes, which are not freely circulating, were mainly studied in the context of embryonic development and wound healing (Ratheesh et al., 2015; Vlisidou and Wood, 2015; Wood and Martin, 2017). Both plasmatocytes and crystal cells have been identified in the embryo, but no lamellocytes (Fossett et al., 2003). Comparing the transcriptomes of embryonic and larval hemocytes, Cattenoz et al., 2020 found that extracellular matrix components were more highly expressed in the former and phagocytosis receptors in the latter. Adults have relatively few hemocytes, and the number is decreasing with age. It has been debated if they are mitotically active. (Ghosh et al., 2015) identified active hematopoiesis in hemocyte clusters in the dorsal abdomen of the fly, but that finding was later refuted by Sanchez Bosch et al., 2019. Recently, Boulet et al., 2021 investigated the hemocyte populations in adult flies using a set of transgenic marker constructs. They reported that mitosis in adult hemocytes is rare and restricted to a separate population of progenitor cells, as discussed in the section about primocytes. Like in the embryo, adult hemocytes are mainly sessile, and their overall transcriptome is more similar to that of embryonic than larval hemocytes. A majority of the adult hemocytes are plasmatocytes and only a small number are crystal cells (Kurucz et al., 2007b).

### Terminology

Rizki’s original terminology, which we have adhered to here, is well established in the *Drosophila* literature. However, we should warn the readers that the plasmatocytes in *Drosophila* should not be confused with the hemocytes called plasmatocytes in other insect orders (Table 1). Instead, *Drosophila* plasmatocytes most likely correspond to the cells called granular cells or granulocytes. In Lepidoptera, granular cells are functional phagocytes, whereas lepidopteran plasmatocytes are main capsule-forming hemocytes, much like *Drosophila* lamellocytes (Strand, 2008). Furthermore, crystal cells and lamellocytes are uniquely found only in the closest relatives of *D. melanogaster*. As discussed in detail below, the crystal cells are homologous to the cells called oenocytoids in other insects. Like crystal cells, oenocytoids are carriers of the phenoloxidase cascade components, but the crystal cell morphology is unique to a few *Drosophila* species. This is all rather confusing, and there is certainly room for a revision of *Drosophila* blood cell terminology.

**Table 1.** Insect hemocyte terminology.

Function	*Drosophila melanogaster* (and related ‘oriental’ subgroup species)	Other drosophilids	Mosquitoes	Lepidopterans(similar terminology in other insect orders)
Phagocytes	Plasmatocytes	Plasmatocytes	Granulocytes/ granular cells	Granulocytes/ granular cells
Melanization	Crystal cells	Oenocytoids (commonly called ‘crystal cells,’ but they lack crystals)	Oencytoids	Oencytoids
Encapsulation	Lamellocytes	Other encapsulating cell types: Nematocytes Multinucleated giant hemocytes Pseudopodocytes Activated plasmatocytes	—	Plasmatocytes (not homologous to *Drosophila* plasmatocytes – uncertain homology to *Drosophila* lamellocytes, as discussed in the text)
?	Primocytes (novel class)	?	?	?
Cuticle formation?	—	—	—	Spherule cells
Hemocyte precursors	Prohemocytes (In lymph gland; few, if any, in circulation)	?	Prohemocytes	Prohemocytes (in hematopoietic organ only?)

The motile form of plasmatocytes seen in *Drosophila* embryos has often been called ‘macrophages,’ and sometimes the same term has been extended to include all plasmatocytes, or even all hemocytes in general. Lanot et al., 2001 and Meister and Lagueux, 2003 used the term to describe the activated plasmatocytes that are observed at the onset of metamorphosis and in the pupa. In this review, we have avoided the macrophage terminology entirely as it could be misunderstood to imply homology (rather that analogy) between *Drosophila* plasmatocytes and vertebrate macrophages. For similar reasons, we here use the term granular cell rather than granulocyte. The specialization of vertebrate blood cells into myeloid and lymphoid lineages probably happened after the split between protostomes and deuterostomes (such as insects and vertebrates, respectively), and the further specialization of vertebrate myeloid cells into macrophages and other subclasses must be an even later event. Nevertheless, specialized phagocytes must have existed throughout metazoan evolution, and plasmatocytes are therefore good models to understand mammalian phagocytes, such as neutrophils, monocytes, dendritic cells, and macrophages.

### *Drosophila* hematopoiesis

During development, hemocytes are produced in two waves (Holz et al., 2003). The first wave is initiated in the embryo, from cells originating in the head mesoderm. These cells give rise to embryonic plasmatocytes and crystal cells, which are then directly carried over to the larvae where they act as founders of the larval circulating and sessile hemocytes. Hemocytes of this first wave also contribute to the pupal and adult hemocyte populations. The second wave originates from the thoracic mesoderm, which develops into a hematopoietic organ situated next to the anterior end of the dorsal vessel in the larva. This hematopoietic organ has been given the unfortunate name ‘lymph gland,’ although its function is more akin to that of the mammalian bone marrow than to lymph glands. Hemocytes are released from the lymph gland at the end of the larval stage, and these hemocytes contribute to the pupal and adult hemocyte populations. In response to parasitoid wasp infection, the lymph gland can also release hemocytes precociously. Cells from both hematopoietic waves contribute to all three classes of hemocytes, plasmatocytes, crystal cells, and, when required, lamellocytes.

### The lymph gland

The genetic control of hematopoiesis has been studied in great detail in the lymph gland. The gland is made up of paired lobes, arranged on each side of the dorsal vessel. The anterior, or primary, lobes are largest and the ones that differentiate first. They are followed by more posterior pairs, the secondary, tertiary, and sometimes quaternary lobes, plus sometimes a variable number of smaller aggregates of hematopoietic cells along the dorsal vessel. The ordered structure of the primary lobes, where cells at different stages of differentiation are organized in different layers, has made them a favorite object of study. Undifferentiated progenitor cells are aggregated in a medially located medulla, usually called the *medullary zone*, which is directly attached to the dorsal vessel. More laterally, differentiating cells form a cortex, the *cortical zone*. Cells in transition are found in an *intermediate zone*, positioned between the medullary and cortical zones.

Finally, a small group of cells at the posterior tip of the primary lobe, in direct contact with the medullary zone, form an interesting and rather mysterious structure, the *posterior signaling center* (PSC). This center was proposed to act as a niche that controls hematopoietic events (Lebestky et al., 2003), an idea that was further supported by the finding that the signaling molecule Hedgehog, secreted from the center, suppresses hemocyte differentiation. In this way, the PSC was suggested to control the balance between undifferentiated precursor cells and differentiating hemocytes (Mandal et al., 2007; Krzemień et al., 2007). However, this interpretation was later challenged by the finding that the proportion of progenitors was unaffected when the PSC was ablated by induced apoptosis (Benmimoun et al., 2015b; Benmimoun et al., 2015a). More complex models have therefore been proposed based on the observation that the medullary zone cells are phenotypically and functionally heterogeneous (Oyallon et al., 2016; Baldeosingh et al., 2018; Banerjee et al., 2019). According to these models, stem cell maintenance is controlled PSC-independently in one subpopulation of medullary zone cells, called core progenitors, PSC-independent progenitors, or preprogenitors. These core progenitors may be precursors of the remaining cells in the medullary zone, the further differentiation of which is controlled by Hedgehog from the PSC. A new twist to this conundrum comes from the recent discovery that the core progenitor population is instead controlled by signals from the dorsal vessel. The dorsal vessel secretes a fibroblast growth factor (FGF) homolog, Breathless, which promotes stem cell maintenance (Destalminil-Letourneau et al., 2021). The PSC itself is also controlled by signals from the dorsal vessel, via a secreted glycoprotein encoded by the *slit* gene. This would all make the dorsal vessel more akin to the vascular hematopoietic niche in vertebrates, while the role of the PSC is more complex.

The posterior signaling center is also required for the induction of lamellocyte formation, independently of its role in stem cell maintenance. Lamellocytes fail to differentiate in *knot* mutant lymph glands, which lack a posterior signaling center (Crozatier et al., 2004), or when PSC cells are ablated by induced apoptosis (Benmimoun et al., 2015b) (*knot* [Bridges and Brehme, 1944] is often referred to by the junior synonym *collier*). Interestingly, these manipulations abolish lamellocyte formation altogether, even among the circulating descendants of the first hematopoietic wave. This indicates that the posterior signaling center either acts remotely, via diffusible signals, or that *knot*-dependent PSC-like cells may exist elsewhere, in direct contact with the peripheral hemocytes. As discussed below, in the section about *primocytes*, the possible existence of such a class of cells is now supported by recent single-cell sequencing data (Cattenoz et al., 2020; Tattikota et al., 2020; Fu et al., 2020).

Unlike the primary lymph gland lobes, the posterior lobes lack a clearly stratified structure, and their hematopoiesis is less well studied. They are not in direct contact with a signaling center, remain undifferentiated at the onset of metamorphosis, and do not initiate differentiation when the animal is infected (Rodrigues et al., 2021).

### Peripheral hematopoiesis

Compared to the orderly events that go on in the lymph gland, hematopoiesis has been more difficult to study in the circulating and sessile larval hemocytes. It is clear, however, that the fully differentiated plasmatocytes, which derive from the first wave of embryonic hematopoiesis, are actively dividing throughout larval development (Makhijani et al., 2011). Mitotic plasmatocytes have been observed both among the freely circulating hemocytes and in the population of sessile hemocytes (Rizki, 1957; Márkus et al., 2009; Kurucz et al., 2007a; Makhijani et al., 2011), and the mitotic activity is highest in connection with each larval molt (Rizki, 1957). Thus, the larval plasmatocytes are propagated by self-renewal of differentiated cells, without contribution from undifferentiated hematopoietic cells in the lymph gland or elsewhere (Makhijani et al., 2011), at least in healthy larvae.

Only about 50% of the larval hemocytes circulate freely in the hemolymph. The remaining cells are attached to the basal membrane under the skin and on other tissues. Circulating and sessile hemocytes are in constant exchange, and the sessile hemocytes can rapidly be mobilized when the animal is disturbed. The attachment of sessile hemocytes depends on the interaction between the membrane protein Eater on the hemocytes and the specialized collagen Multiplexin in the extracellular matrix (Bretscher et al., 2015; Csordás et al., 2020). Under the epidermis, the attachment sites are arranged segmentally in a manner that is regulated by activin-β, secreted by sensory nerves of the peripheral nervous system (Makhijani et al., 2017).

Unlike plasmatocytes, mature crystal cells have never been observed to divide (Rizki, 1957; Leitão and Sucena, 2015). Instead, they are generated by transdifferentiation of fully differentiated plasmatocytes in the sessile compartment (Leitão and Sucena, 2015). This process requires Notch expressed in the transdifferentiating cell, and the Notch ligand Serrate in its plasmatocyte neighbors. The exact role of the sessile compartment in this context is still uncertain. Crystal cells are formed even in *eater* mutant animals, which have no sessile compartment (Bretscher et al., 2015).

Finally, the origin of lamellocytes is not yet entirely settled. Rizki, 1957 proposed that lamellocytes originate from plasmatocytes in the circulating compartment via podocytes as an intermediate stage. Later, Lanot et al., 2001 showed that lamellocytes are formed inside the lymph glands, and they proposed that this is the major, if not the only, source of lamellocytes. This conclusion was in turn questioned by Márkus et al., 2009, who showed that the lymph gland was not required for lamellocyte production. Using a ligation technique, they separated hemocytes in the posterior end from the lymph glands in the anterior part. Wasp infection in the posterior end of the animal triggered lamellocyte formation and encapsulation of the parasite in that part, but not in the anterior half. Furthermore, fluorescently marked sessile cells from the posterior end of a larva gave rise to lamellocytes when they were transplanted into an unmarked host. The present consensus is that both the pre-existing larval hemocyte population and the lymph glands contribute to produce lamellocytes. This conclusion was confirmed by a lineage-tracing approach, showing that lamellocytes in a wasp-infected larva have a mixed origin, including cells from both developmental waves of hematopoiesis (Honti et al., 2010). Notably, the lymph gland-derived lamellocytes were relatively few in this experiment (8% of all lamellocytes), and they were not released into circulation until 2–3 days after infection. Further lineage-tracing experiments have shown that lamellocytes can be generated directly by transdifferentiation of differentiated plasmatocytes (Stofanko et al., 2010; Avet-Rochex et al., 2010).

Recently, a more detailed analysis of the circulating hemocyte population after wasp infection added some complication to this picture (Anderl et al., 2016). At 8–10 hr after infection, a new population of hemocytes, dubbed *lamelloblasts*, was first observed. They were morphologically similar to plasmatocytes, but they were distinguished by a 10-fold lower expression of the plasmatocyte marker, eaterGFP. By 14 hr, the lamelloblasts had increased in number, to become even more abundant than the plasmatocytes. Later, the lamelloblast population was gradually replaced by cells that expressed increasing levels of a lamellocyte marker, msnCherry, and decreasing levels of eaterGFP. These prelamellocytes were finally replaced by fully differentiated msnCherry+, eaterGFP- lamellocytes. Because few intermediates were seen between the plasmatocytes and the lamelloblasts, it was speculated that the lamelloblasts originate from the sessile population. Simultaneously with the changes in the lamellocyte lineage, the plasmatocyte population also changed in appearance, the plasmatocytes became larger and more granular. Later they also began to accumulate cytoplasmic msnCherry-positive foci, suggesting that they had phagocytized lamellocyte fragments. There was evidence of intense mitotic activity among the lamelloblasts and prelamellocytes, but the mature lamellocytes have never been observed to divide (Rizki, 1957). The majority of lamellocytes generated in this way (type I) show no trace of plasmatocyte markers. However, under some circumstances lamellocytes can develop directly from differentiated plasmatocytes, for instance, those attached on the parasite egg (Anderl et al., 2016) or when activated in vitro (Stofanko et al., 2010), resulting in ‘double-positive’ lamellocytes, expressing both plasmatocyte and lamellocyte markers (type II).

### Hematopoiesis in other insects

Historically, a rich literature has described hemocytes from various insect orders (e.g., Cuénot, 1891; Paillot, 1933; Jones, 1962; Lackie, 1988), but for most of them we have little information about their hematopoiesis. Best studied are some mosquitoes and lepidopterans (Strand, 2008).

Compared to the organized structure of the hematopoietic organs in *Drosophila* and *Lepidoptera*, no structured hematopoietic tissue has yet been described in *mosquitoes*. Three main hemocyte types – granulocytes, oenocytoids, and prohemocytes – were distinguished from one another by a combination of morphological and functional markers in two compartments, the circulation and the sessile tissue (Strand, 2008). The hemocytes of the adult females received more attention than those of the larvae as they are vectors of pathogens. However, adult males, pupae, and larvae contain the same hemocyte types as adult females. The sessile hemocytes, in the form of aggregates, however, show different characteristic spatial distribution in different developmental stages (Castillo et al., 2006; Hillyer and Christensen, 2002; League and Hillyer, 2016; League et al., 2017). These aggregates are reminiscent of niches for hemocyte development in lepidopterans and *Drosophila*, suggesting the existence of a dedicated hematopoietic tissue. However, the development of specific markers corresponding to functionally different subsets will be required to characterize the possible functional heterogeneity in these aggregates and help to reveal lineage relationships in specific sites of hematopoiesis.

Studies on *lepidopterans* mainly focus on the immune systems of *Manduca sexta* and *Bombyx mori*, whose larvae contain four, functionally different hemocyte classes: capsule-forming plasmatocytes, phagocytic granular cells, oenocytoids, providing enzymes for the melanization cascade, and spherule cells, with a so far unknown function (Strand, 2008). Similar to the situation in *Drosophila*, the differentiated hemocytes derive both from the embryonic head mesoderm and from specialized hematopoietic organs that are associated with the wing discs in the larva (Nardi, 2004). The lobes of the hematopoietic organ contain prohemocytes and plasmatocytes, whereas the other cell types may derive directly from hemocytes in the circulation (Lavine and Strand, 2002; Strand, 2008). In the hematopoietic organ of *B. mori*, compact and loose regions as well as free cells were observed. The compact islets consist of proliferating prohemocytes and plasmatocytes, whereas differentiated hemocytes are found in the loose regions in late larvae (Grigorian and Hartenstein, 2013). This observation suggests that there is an anatomical and functional subdivision of the organ. In vivo and in vitro analysis of *B. mori* hemocytes confirms that the hematopoietic organ may serve as a niche for hemocyte development in lepidopterans (Nakahara et al., 2010). A comprehensive analysis of the fine structure of the *M. sexta* lymph gland (von Bredow et al., 2021) with a combination of monoclonal antibodies and the lectin peanut agglutinin (PNA) revealed zones with different binding characteristics, showing that the organ is subdivided into anatomical areas with prohemocytes, and differentiating and mature hemocytes, reflecting/suggesting a gradual development of hemocyte subsets within the organ. Ablation experiments revealed that the hematopoietic organ serves as a source of plasmatocytes and putative prohemocytes. However, unlike in *Drosophila*, this occurs throughout the larval stages, not only at the onset of metamorphosis. The lobes of the hematopoietic organs are compartmentalized, but a focus that directs hemocyte development, like the posterior signaling center does in *Drosophila*, has not been observed yet. The relative role of hematopoietic organs and versus hemocytes of embryonic origin as precursors of differentiated hemocytes, and the possible role of transdifferentiation, is still under active investigation (Nardi, 2004; Grigorian and Hartenstein, 2013; Nakahara et al., 2010; von Bredow et al., 2021).

## Single-cell RNA sequencing defines hemocyte heterogeneity

**Figure 1.**
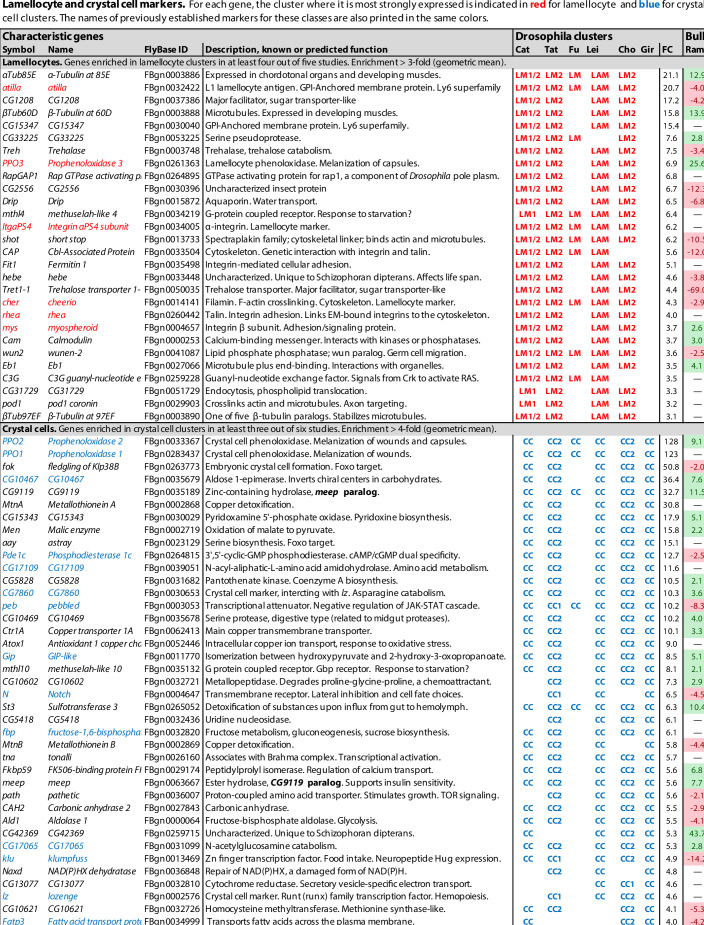
Lamellocyte and crystal cell marker genes. Genes with enhanced expression in lamellocyte- and crystal cell-related clusters, as reported by Cattenoz et al., 2020 (Cat), Tattikota et al., 2020 (Tat), Fu et al., 2020 (Fu), Leitão et al., 2020 (Lei), Cho et al., 2020 (Cho), and Girard et al., 2021 (Gir). Relative expression (‘FC’) in bulk plasmatocytes compared to whole larvae, as reported by Ramond et al., 2020, is shown in a separate column (Ram). The figure summarizes the most consistently and strongly enhanced genes for each of these cell classes, and the average (geometric mean) fold enhancement (‘FC’). As we lack full data from Fu, we have only listed examples mentioned in the text and figures of that study. For a full list of all enhanced genes, see Figure 1—source data 1. Figure 1—source data 1.Genes with enhanced expression in specific cell classes.Complete lists of differentially expressed genes. Figure 1—source data 2.Gene Ontology (GO) terms.Significantly enriched GO terms in differentially expressed genes in lamellocyte, crystal cell, and plasmatocyte clusters in at least two of the published studies.

**Figure 2.**
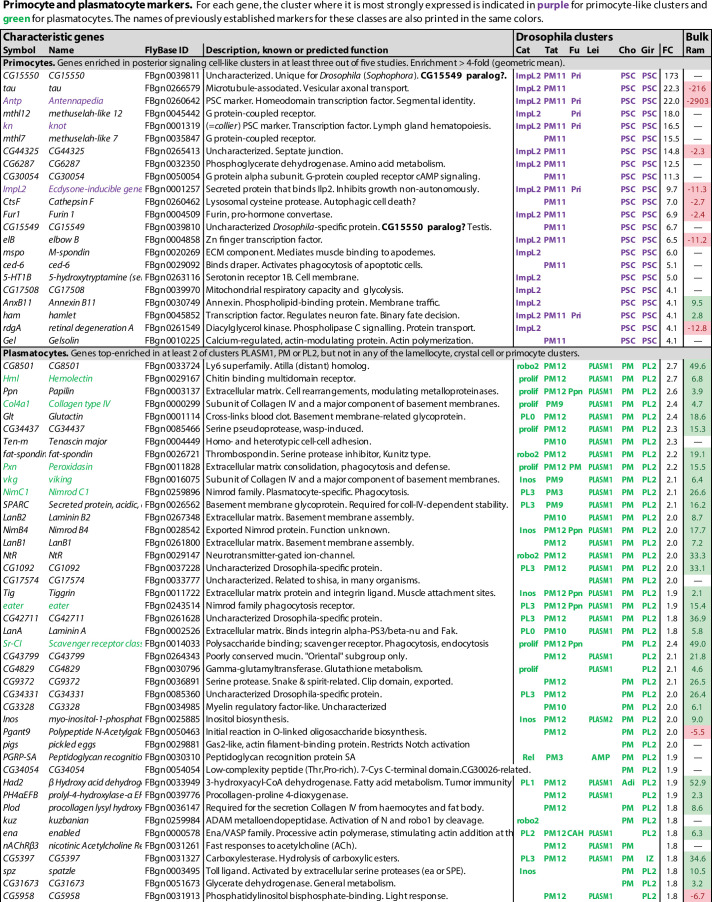
Primocyte and plasmatocyte marker genes. Genes with enhanced expression in primocyte- and plasmatocyte-related clusters. Details as in Figure 1.

Recently, several groups have used single-cell RNA sequencing technology to study the hemocyte diversity in *Drosophila* larvae and the relationship between the different hemocyte classes. Four published studies deal with the peripheral hemocytes, that is, the sessile and circulating hemocytes of the first hematopoietic wave (Cattenoz et al., 2020; Tattikota et al., 2020; Fu et al., 2020; Leitão et al., 2020), and two focus on the lymph glands (Cho et al., 2020; Girard et al., 2021). The Fly Cell Atlas, which appeared recently (Li et al., 2022), presents additional data on all cells in the adult fly, including hemocytes. These studies have confirmed the existence of unique hemocyte ‘clusters,’ corresponding to the classically defined crystal cell and lamellocyte classes, and in some cases also to their precursors. Not surprisingly, however, the plasmatocytes turned out to be heterogenous, and they were split by the different authors into several different clusters. Each cluster was defined by a unique pattern of gene expression, but these patterns were not entirely congruent between the different studies. The six studies on larval hemocytes identified between 2 and 13 plasmatocyte clusters, and in addition between 3 and 6 prohemocyte clusters in the lymph glands. A consensus view of the situation was recently published (Cattenoz et al., 2021), drawing general conclusions from three of the studies (Cattenoz et al., 2020; Tattikota et al., 2020; Fu et al., 2020). To bring further clarity to this issue, we have now compiled lists of the genes that are specifically expressed in each cluster and compared these lists from the different studies (Figure 1—source data 1). To reduce noise, we set a threshold of at least 1.4-fold enrichment (2-fold for the data from Girard et al., 2021, where the relative enrichment values were generally higher). As Fu et al., 2020. did not provide comprehensive lists of specifically expressed genes, we have only listed genes mentioned in the text and figures. The most characteristic lamellocyte and crystal cell markers are listed in Figure 1, and primocyte and plasmatocyte markers in Figure 2.

**Figure 1—figure supplement 1.**
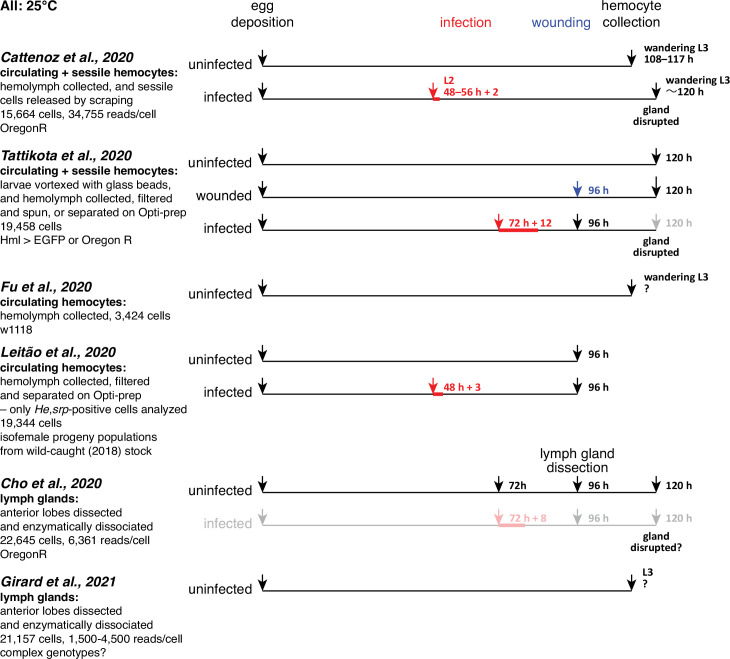
Timelines for six single-cell transcriptomic analyses of *Drosophila* hemocytes. Data from the analyses shown in gray were not included in our comparisons.

**Figure 2—figure supplement 1.**
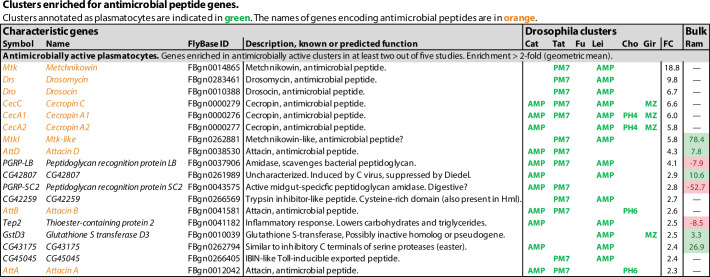
Clusters enriched for antimicrobial peptide genes. Genes with enhanced expression in the AMP clusters, as reported by Cattenoz et al., 2020 (Cat), and Leitão et al., 2020 (Lei), and the PM7 cluster of Tattikota et al., 2020 (Tat). Clusters where the same genes are enriched as reported by Cho et al., 2020 (Cho) and Girard et al., 2021 (Gir) are also indicated. None of these genes were mentioned by Fu et al., 2020 (Fu). Relative expression (‘FC’) in bulk plasmatocytes compared to whole larvae, as reported by Ramond et al., 2020, is shown in a separate column (Ram). The figure summarizes the most consistently and strongly enhanced genes for each of these cell classes, and the average (geometric mean) fold enhancement (‘FC’). For a full list of all enhanced genes, see Figure 1—source data 1.

### Lamellocytes: Highly active immune effector cells

In total, transcripts of 136 genes were significantly enriched in lamellocyte clusters in at least two of the studies, and 32 were enriched in at least four studies (Figure 1, Figure 1—source data 1). These genes include the well-established lamellocyte markers *atilla*, *PPO3*, *ItgaPS4*, *cheerio*, *rhea* (*talin*), and *mys* (Kurucz et al., 2007b; Dudzic et al., 2015; Irving et al., 2005). The widely used lamellocyte marker *misshapen* (*msn*) (Tokusumi et al., 2009a) turned up in only one of the studies (Tattikota et al., 2020). Disappointingly, these lamellocyte markers were not exclusively detected in the lamellocyte clusters only. Only five genes were consistently enriched by 10-fold or more, while other marker genes were typically enriched 4-fold or less. This could be due to incomplete separation between the lamellocyte clusters and other hemocytes, to admixture with lamellocyte precursors, or to uptake of lamellocyte fragments by other cells as discussed below.

The highly expressed genes *atilla* and *CG15347* both encode GPI-anchored membrane proteins, related to a group of Ly6-like proteins that mediate septate junction formation (Nilton et al., 2010). They may play a role in strengthening the capsules formed by these effector cells around parasites, while the *Prophenoloxidase 3* (*PPO3*) gene encodes a phenoloxidase that is involved in the melanization of these capsules (Nam et al., 2008; Dudzic et al., 2015).

Strikingly, the list of lamellocyte-specific genes includes many genes involved in cytoskeletal activity, cell adhesion, cell motility, and even muscle activity (Figure 1—source data 2), suggesting a physically very active role for these cells. Two tubulin genes, *αTub85E* and *βTub60D*, are among the most highly enriched transcripts in the lamellocyte clusters, and two others, *αTub84B* and *βTub97EF,* are also on the list. Furthermore, two cytoplasmic actin genes, *Act42A* and *Act5C*, and no less than 21 genes involved in actin filament-based processes are more or less enriched in at least two of the five studies (Figure 1—source data 2). Regarding genes involved in cell adhesion, two α-integrins, *ItgaPS4* and *mew*, and two β-integrins, *mys* and *Itgbn,* are highly enriched, as well as several components of the intracellular machinery that mediate integrin interaction with the cytoskeleton, and integrin-mediated cell adhesion: *rhea*, *plx*, *parvin*, *stck*, *Pax*, *ics,* and *Ilk*.

In line with a physically active role for the lamellocytes, the most highly enriched genes include the *Trehalase* (*Treh*) and *Trehalose transporter 1-1* (*Tret1-1*) genes (Figure 1), which mediate the uptake and utilization of trehalose from the hemolymph as an energy source for the cells. *CG1208* encodes another potential sugar transporter that may also be involved in this traffic. As shown by Bajgar et al., 2015, the uptake of sugars into hemocytes is dramatically increased in wasp-infected larvae. Other tissues, muscles in particular, respond by mobilizing glycogen stores in order to supply trehalose to the hemolymph (Yang and Hultmark, 2017). The up to 30-fold increased expression of sugar transporters in lamellocytes, compared to other hemocytes (Figure 1), suggests that the lamellocytes are major consumers of these sugars. Cattenoz et al., 2021 also noted that target genes of Tor and foxo, which regulate nutrient metabolism, were particularly enriched in the lamellocyte clusters. This is most likely connected to extreme metabolic needs in these cells.

The geared-up metabolism in the lamellocytes may also be associated with a switch towards aerobic glycolysis in the lamellocytes, mediated by extracellular adenosine released from immune cells (Bajgar et al., 2015), although that metabolic switch has been best studied in phagocytically activated plasmatocytes (Bajgar and Dolezal, 2018; Krejčová et al., 2019; Bajgar et al., 2021). However, in agreement with a specific role of extracellular adenosine in lamellocyte hematopoiesis, the *adenosine deaminase-related growth factor A* (*Adgf-A*) and *adenosine receptor* (*AdoR*) transcripts are enriched in the lamellocytes (Figure 1—source data 1). *AdoR* encodes a G protein-coupled receptor that functions via cAMP and PKA activation. Accordingly, target genes for the cyclic-AMP response element binding protein B (CrebB) are also enriched in the lamellocytes (Cattenoz et al., 2021). *Adgf-A* encodes a deaminase that regulates the level of extracellular adenosine.

Finally, target genes of the JNK pathway are also enriched in the lamellocyte clusters (Cattenoz et al., 2021). This supports the idea that JNK signaling may be directly involved in lamellocyte differentiation (Zettervall et al., 2004; Tokusumi et al., 2009b).

### Crystal cells

**Figure 3.**
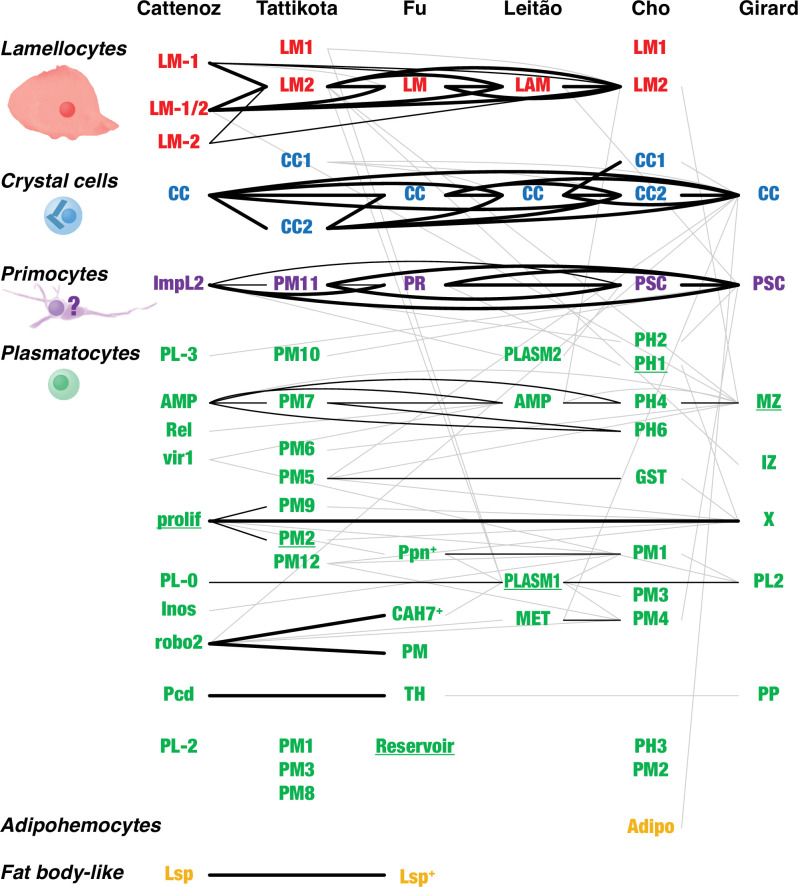
Overlap between *Drosophila* hemocyte clusters. Lines connect clusters that share genes with >1.4-fold enriched transcripts. A fat line indicates that at least 50% of the genes enriched in one of the clusters are also enriched in the other one (but not necessarily reciprocally). A thin line indicates that both clusters share at least 10% of the enriched genes. A thin gray line indicates that one cluster shares 10% of the enriched genes with the other one, but not reciprocally. The different clusters were named after their presumed identity: lamellocytes (LM, LAM), crystal cells (CC), primocytes (PR), plasmatocytes (PL, PM, PLASM), prohemocytes (PH), proplasmatocytes (PP), medullary zone cells (MZ), intermediate zone cells (IZ), posterior signaling center (PSC), or after their presumed functions: metabolic (MET), reservoir. The clusters prolif and X represent mitotic cells. Some plasmatocyte clusters were named after characteristic genes or gene groups that are enriched in the clusters (ImpL2, AMP, Rel, vir1, Inos, robo2, Pcd, Lsp, Ppn, CAH7, GST). Two additional suggested classes, thanacytes (TH) and adipohemocytes (Adipo), were not reproducibly observed and are not further discussed here. Finally, no genes were preferentially expressed above our cutoff in clusters PL-1 (Cattenoz et al., 2020), PM4, and PM11 (Tattikota et al., 2020). The shape and size of circulating larval primocytes are unknown. Instead, the illustration is based on published images of primocyte-like cells in adults (Boulet et al., 2021) and primocytes in the posterior signaling center (Krzemień et al., 2007; Mandal et al., 2007).

The crystal cell clusters also form a well-defined class, in this case with a high degree of overlap between all six studies (Figure 3). 137 genes were preferentially expressed in crystal cell clusters in at least two of the studies and 35 genes in at least five of them (Figure 1, Figure 1—source data 1). However, the level of enrichment varied enormously between the studies, with particularly high values reported by Girard et al., 2021. The putative precursors in the CC1 clusters (Tattikota et al., 2020; Cho et al., 2020) overlap less with the major crystal cell clusters.

As expected, well-established crystal cell markers like *PPO1*, *PPO2* (Binggeli et al., 2014), and *lozenge* (*lz*) (Ferjoux et al., 2007) were represented among the preferentially expressed genes, although high enrichment of *lozenge* (53-fold) was reported in one study only. In the other studies, *lozenge* was enriched threefold at best, if at all (Figure 1, Figure 1—source data 1). By contrast, the phenoloxidase genes *PPO1* and *PPO2* were very highly enriched (up to 836-fold) in all six studies. *PPO3* has also been used as a crystal cell marker, but only in the embryo (Waltzer et al., 2003; Bataillé et al., 2005; Ferjoux et al., 2007), but that gene was exclusively lamellocyte-specific in the studies discussed here. More rarely used crystal cell markers like *peb*, *klu* (Terriente-Felix et al., 2013) and *Jafrac1* (Waltzer et al., 2003) were also overrepresented in the crystal cell clusters, as were 19 of 31 embryonic crystal cell markers listed by Ferjoux et al., 2007: *PPO1*, *PPO2*, *CG10467*, *Pde1c*, *CG17109*, *Gip*, *fbp*, *CG17065*, *Cndp2*, *CG31431*, *Jafrac1*, *Fatp3*, *Kaz-m1*, *CG1847*, *ApepP*, *pfrx*, *CG15739*, *Rift,* and *CG8112* (Figure 1, Figure 1—source data 1). A Gene Ontology (GO) term analysis of genes specifically enriched in crystal cells in at least two of the studies shows that genes involved in basal metabolism are highly enriched (Figure 1—source data 2), suggesting that crystal cells are metabolically very active.

Crystal cells are best known for their role in the melanization of wounds and encapsulated parasites, as reflected in the list of genes that are preferentially expressed in these cells (Figure 1). *PPO1* and *PPO2* encode phenoloxidases, key enzymes in the melanization reaction. They are copper enzymes that catalyze the oxidation of tyrosine and other phenols, leading to their polymerization into melanin (Carton et al., 2008). PPO1 is produced in an inactive form, prophenoloxidase, which is possibly secreted directly into the hemolymph. By contrast, PPO2 is kept sequestered in the crystals and released only when the activated crystal cells rupture and the crystals dissolve (Binggeli et al., 2014; Schmid et al., 2019). The proenzymes are proteolytically processed in the hemolymph into active phenoloxidase forms, in a process that involves several serine proteases (Nam et al., 2012; Dudzic et al., 2019). Together, PPO1 and PPO2 mediate the melanization of wound sites and of infecting bacteria (Binggeli et al., 2014; Dudzic et al., 2019). PPO2 from crystal cells also acts together with PPO3 in lamellocytes to melanize encapsulated parasites (Dudzic et al., 2015). Related to the production of these copper enzymes, the transcripts for two copper-transporting and -concentrating proteins, encoded by the *Ctr1A* and *Atox1* genes, are overrepresented in the crystal cells. Four metallothionein genes are also specifically transcribed in the crystal cells: *MtnA*, *MtnB*, *MtnD,* and *MtnE* (Figure 1, Figure 1—source data 1). Their role may be to deal with copper toxicity.

The capacity of crystal cells to burst and release their contents, via a pyroptosis-like mechanism (Dziedziech and Theopold, 2021), in response to infection and other challenges, may also be reflected in the single-cell transcriptome data. In all six studies, *Ninjurin B* (*NijB*) transcripts were enriched in the crystal cell clusters (Figure 1—source data 1). *NijB* encodes a homolog of the human Ninjurin-1, which is described as a cell adhesion protein. The corresponding mouse protein, NINJ1, was recently found to mediate plasma membrane rupture in macrophages, in response to bacterial infection, and thereby the release of pro-inflammatory cytokine IL-1β and other danger signals (Kayagaki et al., 2021). *NijB* may play a similar role in the infection-induced rupture of crystal cells.

The membrane receptor Notch plays a central role in crystal cell fate determination and maintenance, together with the Runt domain transcription factor Lozenge, which acts downstream and together with Notch. Consequently, *Notch* (*N*) and *lozenge* (*lz*) transcripts were picked up in most of the studies as preferentially expressed in the crystal cells, as were several Notch and Lozenge transcriptional targets: the early-onset Notch targets *E(spl)m3-HLH* and *E(spl)mβ-HLH* (Couturier et al., 2019), which encode basic helix–loop–helix transcription factors; and the Notch/Lozenge target genes *pebbled* (peb = *hindsight*); *klumpfuss* (*klu*); and *CG32369* (Terriente-Felix et al., 2013). Transcripts of the *numb* gene, which encodes a membrane-associated inhibitor of signaling from Notch (Wu and Li, 2015), were also found to be enriched, but only in the lymph gland studies.

The interpretation of the crystal cell transcriptome data is complicated by the fact that the recovery of crystal cells was in some cases very low. While normally about 5–10% of the hemocyte population are crystal cells in uninfected third-instar larvae, only 0.6% (Cattenoz et al., 2020) or 0.35% (Fu et al., 2020) of all counted hemocytes were assigned to the crystal cell clusters. Although other studies found higher numbers, many cells may have been lost due to the sensitive nature of crystal cells, which tend to burst after bleeding.

### Primocytes: A new class of hemocytes related to the cells of the posterior signaling center

Unexpectedly, one additional well-defined hemocyte class was standing out in these comparisons, besides lamellocytes and crystal cells. Clusters belonging to this class have a pattern of gene expression that is indistinguishable from cells of the posterior signaling center of the lymph glands. This cluster was called PL-ImpL2 by Cattenoz et al., 2020, who also noted the similarity to the posterior signaling center (Cattenoz et al., 2021), PM11 by Tattikota et al., 2020, and primocytes by Fu et al., 2020 (Figure 3). We will here use the term primocytes as an inclusive term for these clusters together with the PSC clusters described from the lymph glands (Cho et al., 2020; Girard et al., 2021; Figure 2, Figure 1—source data 1). Notably, the primocyte class went undetected in the Leitão et al., 2020 study, perhaps because primocytes are relatively rare, only about 0.3% of all peripheral hemocytes (Cattenoz et al., 2020; Fu et al., 2020). Alternatively, they may have been lost in the *He*, *srp*-selection step employed by Leitão et al., if these markers are not expressed by the primocytes.

Of the genes that were preferentially expressed in primocyte clusters, 69 were identified in at least two studies, and 12 in at least four of the six studies (Figure 2, Figure 1—source data 1). Among these genes, one gene, *CG15550*, is uniquely standing out as highly enriched in all primocyte clusters. It encodes a small predicted transmembrane protein of unknown function. *CG15550* has highly conserved homologs among *Drosophila* species in the *melanogaster* and *obscura* groups of the *Sophophora* subgenus, but is strikingly absent in other organisms. Highly enriched are also well-established markers for the posterior signaling center, such as *Antennapedia* and *knot* (*collier*). Another gene that was identified as enriched in the primocyte clusters is *ImpL2*, which encodes a secreted insulin antagonist (Honegger et al., 2008) that can cause wasting by redirecting nutrients to proliferating tissues (Kwon et al., 2015). Bajgar et al., 2021 have shown that ImpL2 is secreted from certain circulating hemocytes, most likely primocytes, and thereby induces adipose tissue to release lipoproteins and carbohydrates that can be utilized by the activated immune system.

The discovery of circulating primocytes may resolve the old question how manipulations that affect the posterior signaling center can control the generation of lamellocytes not only in the lymph gland, but also in the peripheral population of hemocytes. The genetic ablation of primocytes in the posterior signaling center (Crozatier et al., 2004; Benmimoun et al., 2015b) is likely to ablate primocytes also elsewhere. An important function of the primocytes may be to directly trigger lamellocyte formation in the peripheral compartment as well as in the lymph gland, either by direct contact with lamellocyte precursors or via diffusible signals.

The expression of *Antennapedia* (*Antp*) in the posterior signaling center has been taken as evidence that it originates from the mesodermal T3 segment in embryonic development, unlike the primary lymph gland lobes, which arise from segments T1-T2, and the larval hemocytes, which arise from head mesoderm (Mandal et al., 2007). *Antp* is also ubiquitously expressed in the circulating primocytes, suggesting that they may also have a different origin from the other larval hemocytes. It should be investigated if cells are released from the posterior signaling centers, or perhaps from other T3-derived cells, long before the rupture of the lymph gland lobes. It is also worth noting that primocyte-like (i.e., PSC-like) cells have also been detected in at least one posterior lymph gland lobe, the tertiary lobe. Like primocytes, they express *knot* (*collier*), but instead of *Antp* they express a more posterior homeotic gene, *Ubx* (Rodrigues et al., 2021; Kanwal et al., 2021). The exact role of these cells also remains to be investigated.

The circulating primocytes were generally interpreted as a subclass of plasmatocytes (Cattenoz et al., 2020; Tattikota et al., 2020; Fu et al., 2020; Leitão et al., 2020). However, since they probably have a common origin with the posterior signaling center, not with the other hemocyte classes, and since a majority of the primocyte-specific markers are highly depleted or absent in bulk plasmatocytes (Ramond et al., 2020; Figure 2), we will here treat them as a separate class of hemocytes.

In their recent study of adult hemocytes, Boulet et al., 2021 identified a small cell population that expressed the *domeMeso-GAL4* driver, a marker for hemocyte progenitors in the medullary zone of the larval lymph gland (Banerjee et al., 2019), and they concluded that these cells were prohemocytes. However, lineage tracing suggested that they derived from the posterior signaling center (or from a similar primocyte source). Furthermore, these cells expressed primocyte markers such as the *Antp* gene and the *col-GAL4* driver (with the *knot* [*col*] promoter). Thus, this cell population may correspond to bona fide primocytes. These putative primocytes had a fusiform shape, with long filopodial extensions, much like the filopodia that extend from the posterior signaling center into the primary lymph gland lobes (Krzemień et al., 2007; Mandal et al., 2007). This gives them an appearance that is reminiscent of the nematocytes that have been described from other drosophilid species, discussed below.

In conclusion, primocytes constitute a distinct hemocyte lineage with a different origin than other hemocytes. The functional role of the circulating primocytes remains speculative, but it is possible that they interact with and control the peripheral plasmatocytes, like the primocytes of the posterior signaling center control hemocytes in the primary lobe of the lymph gland. That interaction would be facilitated if the circulating larval primocytes are shaped like the putative adult primocytes, with long extensions. However, this interpretation is not in line with the description of the adult primocyte-like cells as a set of prohemocytes with capacity to divide and to differentiate into plasmatocytes (Boulet et al., 2021).

### Plasmatocytes: Multitasking and very plastic cells

The data analyses in the single-cell transcriptomic studies discussed here were primarily designed to identify different plasmatocyte subgroups, not to find common markers for plasmatocytes in general. As a proxy for such pan-plasmatocyte markers, we combined three subclusters that express several classical plasmatocyte markers: (1) the PLASM1 cluster of Leitão et al., 2020, (2) the PM cluster of Cho et al., 2020, and (3) the PL2 cluster of Girard et al., 2021. After weeding out genes that are more strongly expressed in lamellocytes, crystal cells or primocytes in any of the other transcriptome studies, we could assemble a list of 125 putative plasmatocyte-specific marker genes, 46 of which were expressed in at least two of the three clusters (Figure 2, Figure 1—source data 1). This tentative list includes well-known plasmatocyte marker genes such as *Hemolectin*, *Col4a1*, *Peroxidasin*, *viking*, *NimC1*, *eater,* and *Sr-CI* (Figure 2; Goto et al., 2003; Fessler et al., 1994; Paladi and Tepass, 2004; Irving et al., 2005; Kurucz et al., 2007a; Kroeger et al., 2012). However, we can neither be sure if these markers are exclusively expressed in plasmatocytes only, nor if they are ubiquitously expressed in every plasmatocyte. To resolve these questions, raw data will have to be reanalyzed under conditions such that all plasmatocytes fall into one cluster.

It is interesting to compare this list with the bulk transcriptomic analysis of total (*Hemolectin*-positive) plasmatocytes recently published by Ramond et al., 2020, as shown in the last column in Figure 2, although it should be kept in mind that the single-cell data shows the expression in one cluster compared to all other clusters, while the bulk data shows the expression in all (*Hemolectin*-positive) plasmatocytes compared to the total expression in the entire larva. Nevertheless, there is good correlation between the single-cell and the bulk plasmatocyte data sets, except that the relative enhancement is generally much higher in the bulk data, presumably because plasmatocytes are also present in the reference clusters of the single-cell data. Most primocyte markers, like *Antp* and *knot* (*collier*), are strongly depleted or undetected in the plasmatocyte data of Ramond et al., 2020, giving further support to the conclusion that primocytes are unrelated to the plasmatocyte class. Lamellocyte and crystal cell markers also tend to be underrepresented in the bulk plasmatocyte cell data, but there is some overlap, perhaps because the plasmatocyte sample includes precursors of lamellocytes and crystal cells.

Strikingly, a large number of plasmatocyte-specific genes in the list encode basement membrane components, or are involved in extracellular matrix formation or in cell–matrix or cell–cell adhesion (Figure 2, Figure 1—source data 2). We conclude that plasmatocytes must be constantly active in shaping and reshaping the extracellular matrix (Fessler et al., 1994). The list also includes several known or suspected phagocytosis receptors and microbial pattern recognition molecules (Figure 2), such as *NimC1*, *NimB4*, *eater*, *Sr-CI,* and *PGRP-SA* (but not *PGRP-LC*), as well as the lectins *Hemolectin* and *lectin-24Db*. This is in line with a role of plasmatocytes in recognizing and phagocytizing microorganisms.

The subclustering analysis of the single-cell transcriptomic data documented much plasmatocyte heterogeneity (Cattenoz et al., 2020; Tattikota et al., 2020; Fu et al., 2020; Leitão et al., 2020; Cho et al., 2020; Girard et al., 2021), but we find only limited congruence between the different studies (Figure 3). Based on the available data, it is therefore still not possible to identify any well-defined plasmatocyte subclasses. Thus, it may be more practical to treat the plasmatocytes as a single class, albeit a very plastic one, that turns on different transcriptional programs depending on the needs of the moment (Mase et al., 2021). The entire complement of plasmatocytes will then represent a continuum of cells that to a variable extent have activated one or more of these programs.

Subclusters with an activated antimicrobial program were identified in all but one of the published studies (Figure 2—figure supplement 1, Figure 1—source data 1). These clusters include the ones called PL-AMP by Cattenoz et al., 2020, PM7 by Tattikota et al., 2020, AMP by Leitão et al., 2020, PH4 and PH6 by Cho et al., 2020, and MZ by Girard et al., 2021. The overlap between the studies was modest. Only 21 genes, almost all of them known targets of the Imd and/or Toll signaling pathways, were shared by two or more of the studies. Only three genes, encoding different cecropins, were identified in more than three of the studies. Besides these targets of the antimicrobial response, there was very little overlap between the different studies.

Similarly, the PM5 cluster of Tattikota et al., 2020 and the GST cluster of Cho et al., 2020 define a program for oxidative stress. These clusters share only 15 genes, but both clusters include genes involved in the response to oxidative stress, such as different glutathione S transferases (GSTs) (Figure 1—source data 1).

The PL-Pcd cluster of Cattenoz et al., 2020 and the Thanacyte cluster of Fu et al., 2020 have a significant overlap, and together they define cells involved in a program for protein export. These cells specifically express genes involved in the protein export pathways as well as genes encoding exported proteins, notably three thioester-containing proteins (TEPs) (Figure 1—source data 1).

Two of the studies have identified cell clusters that express a mitotic program. The PL-prolif cluster of Cattenoz et al., 2020 and the X cluster of Girard et al., 2021 share a large number of genes involved in the cell cycle (Figure 1—source data 1). There is also a small but significant overlap with the PM2 and PM9 clusters of Tattikota et al., 2020.

The clusters called PL-Lsp (Cattenoz et al., 2020) or Lsp^+^ PM (Fu et al., 2020) constitute a special case. Besides a normal complement of plasmatocyte-specific genes, they are highly enriched for several genes that are otherwise only expressed in the fat body. The fact that they express plasmatocyte markers excludes the possibility that they represent a contamination by fat body cells. It is possible that these cells function as nutrient reservoirs, as suggested by Cattenoz et al., 2020. Alternatively, they may simply be plasmatocytes that have engulfed fat body fragments, in preparation for metamorphosis, as discussed below.

Cattenoz et al., 2021 have made a careful and more detailed comparison between two of the studies (Cattenoz et al., 2020; Tattikota et al., 2020), and also taken the results of Fu et al., 2020 into account. Besides lamellocytes, crystal cells, and PSC-like cells (primocytes), they proposed five subgroups of plasmatocytes: proliferative, antimicrobial, phagocytic, secretory, and unspecified plasmatocytes. That classification scheme is similar to the programs we describe above, although the subgroups defined by Cattenoz et al., 2021 tend to include additional clusters. The difference seems to be due to the lower cutoff values set by Cattenoz et al., 2021. For instance, the ‘proliferative’ subgroup includes not only the PL-prolif cluster but also the PL-Inos cluster of Cattenoz et al., 2020. However, the level of enrichment of mitosis-specific genes is very low in the latter cluster. The most highly enriched mitotic gene, *string*, is 9.7-fold enriched in PL-prolif, but also 1.9-fold in PL-Inos and, surprisingly, 1.3-fold in CC. In the data from Tattikota et al., 2020, it is 1.5-fold enriched in PM9, 1.4-fold in PM2, and 1.2-fold in PM1. We conclude that low levels of mitotic activity may go on in many clusters. In general, plasmatocytes seem to be engaged in many different activities, sometimes simultaneously and to a variable degree. That makes it difficult to classify them into well-defined and reproducible subgroups.

Earlier literature has documented many aspects of plasmatocyte plasticity, and the different roles of plasmatocytes are very well described in a recent review (Mase et al., 2021). At the onset of metamorphosis, plasmatocytes become very active. They become adhesive and motile, take on a *podocyte* morphology, and begin to phagocytize large quantities of histolyzing larval tissue, in particular muscle and fat (Lanot et al., 2001; Meister and Lagueux, 2003; Sampson and Williams, 2012; Ghosh et al., 2020). Major changes have also been observed in the plasmatocytes of wasp-infected larvae. Besides lamellocytes and their precursors, which turn up in the hemolymph of the infected larva, a population of cells of the plasmatocyte lineage begin to increase in size and granularity about 10 hr after infection and such *activated plasmatocytes* become abundant after 30 hr (Anderl et al., 2016). The activated plasmatocytes were observed to express increased levels of the *eaterGFP* plasmatocyte marker, and they also accumulate inclusions that express the *msnCherry* lamellocyte marker, most likely remnants of phagocytized lamellocyte fragments. The changed plasmatocyte activity before metamorphosis and after infection is unfortunately not reflected in the single-cell sequencing studies discussed here, but calls for more extensive time series of infected and uninfected animals.

Beyond the limits of this plasticity, which seems to be largely reversible, plasmatocytes also have a capacity to transdifferentiate irreversibly to become crystal cells and lamellocytes (Leitão and Sucena, 2015; Honti et al., 2010; Avet-Rochex et al., 2010; Stofanko et al., 2010), in the latter case via intermediate stages such as lamelloblasts and prelamellocytes (Anderl et al., 2016). Such intermediate stages are exemplified by the CC1 and LM1 clusters of Tattikota et al., 2020, for crystal cells and lamellocytes, respectively, and the LM-2 prelamellocytes of Cattenoz et al., 2020. It should be noted that these crystal cell and lamellocyte precursor clusters share no genetic markers with the similarly named CC1 and LM1 clusters of Cho et al., 2020, which originate from prohemocytes, not plasmatocytes.

In conclusion, the plasmatocyte subclusters show disappointingly little overlap between the different studies. The described clusters are either unique or share a limited number of enriched genes between just a few of the studies. The only exceptions are clusters involved in an antimicrobial program. Such cells were noted in most of the studies, but the overlap includes only a very narrowly defined class of genes. The general picture is that the plasmatocytes constitute a cell class that serves many tasks, each task requiring the activation of a few specialized genes, without requiring a complete re-differentiation of the cells. Lineage-tracing assays will establish whether the observed specific features are lineage related (identity) or depend on the environment (state), whether specified plasmatocytes arise from the nonspecified ones or from differentiated plasmatocytes that change potential.

### Prohemocytes: Only in the lymph gland

It has been argued that the mitotically active hemocytes in circulation represent a prohemocyte population (Cattenoz et al., 2021), but since they express plasmatocyte markers our tentative interpretation is that mitosis occurs as a transient stage in the life of a plasmatocyte. It is uncertain if prohemocytes, that is, self-renewing and truly undifferentiated hemocytes, ever occur in circulation in *Drosophila*, but in the lymph gland they occupy the medullary zone, and it cannot yet be ruled out that a population of prohemocytes is hiding among the sessile hemocytes in the larva. However, it has been demonstrated that crystal cells are generated by transdifferentiation of fully differentiated sessile plasmatocytes under the skin of the larva (Leitão and Sucena, 2015), and lamellocytes are also generated from the plasmatocyte lineage in the larva (Honti et al., 2010; Avet-Rochex et al., 2010; Stofanko et al., 2010). Anderl et al., 2016 could directly confirm how plasmatocytes that were attached to the egg of a parasitoid wasp transdifferentiate into lamellocytes type II. On the other hand, Anderl et al. also observed that a large population of undifferentiated and self-proliferating hemocytes, the lamelloblasts, appeared in the *Drosophila* larva soon after wasp infection, and these cells later seemed to differentiate into lamellocytes via an intermediate prelamellocyte stage. It is possible that the LM1 clusters of Tattikota et al. and Cho et al., which share few if any markers with the differentiated lamellocyte clusters (Figure 3), correspond to lamelloblasts.

Thus, true prohemocyte clusters were only identified in the lymph gland studies, the prohemocyte clusters: PH1–PH6 of Cho et al., 2020 and the medullary zone cluster MZ of Girard et al., 2021. Surprisingly, these clusters share few markers with each other (or with other hemocyte clusters), except that MZ and PH4 both have enhanced expression of the *CecA1*, *CecA2,* and *CecC* antimicrobial peptide genes. The functional importance of that observation is unclear at the moment. For an update on the interesting field of lymph gland hematopoiesis, interested readers are referred to recent reviews (Banerjee et al., 2019; Csordás et al., 2021; Morin-Poulard et al., 2021).

## Relationship to blood cells in other species

Armed with the new markers for specific cell classes in *D. melanogaster*, we can begin to look for homologous cell types in other species. Beginning with the crystal cells, where the relationships are more clear, we will here discuss the results from three single-cell transcriptomic studies of hemocytes from the malaria mosquito, *Anopheles gambiae*, and one from the silkworm, *B. mori* (Severo et al., 2018; Raddi et al., 2020; Kwon et al., 2021b; Feng et al., 2021). We will also discuss transcriptomic and genomic information available for drosophilid flies other than *D. melanogaster*.

### Crystal cells/oenocytoids

**Figure 4.**
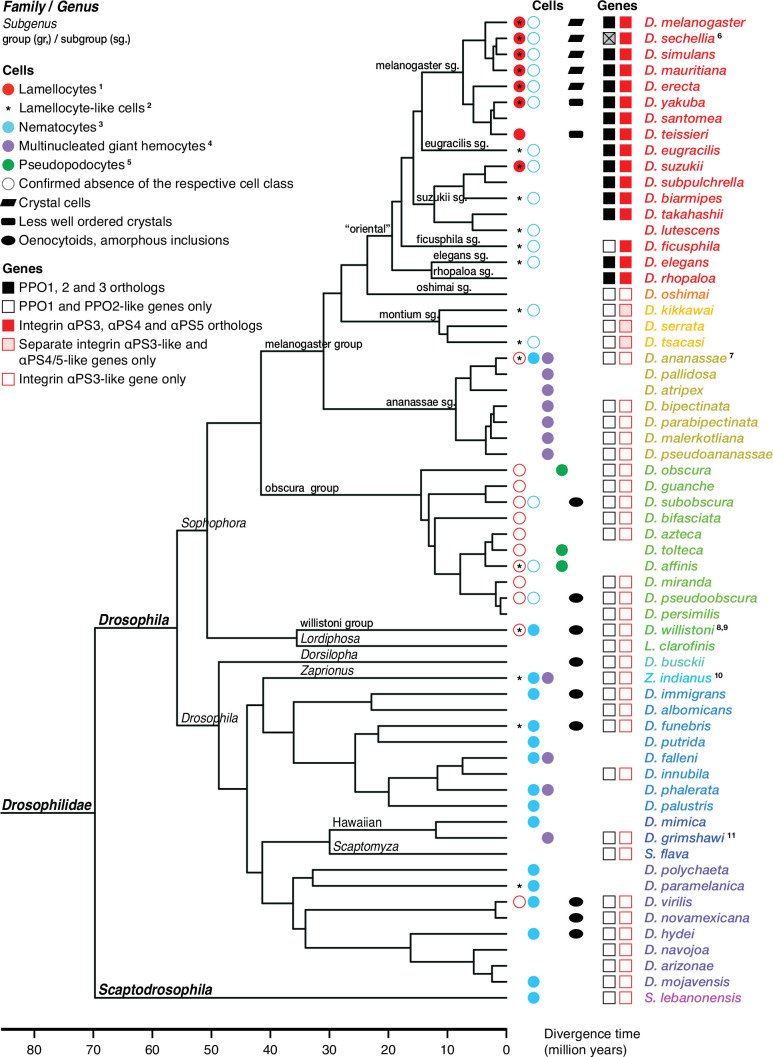
Occurrence of specialized effector cells (lamellocytes, nematocytes, multinucleated giant hemocytes, pseudopodocytes, and crystal cells) in parasitized drosophilid larvae, and correlation with presence or absence of *PPO3* and *ItgaPS4* genes. Consensus phylogenetic tree from Russo et al., 2013, Thomas and Hahn, 2017, Miller et al., 2018, Kim et al., 2021, and Finet et al., 2021. Basic topology from Finet et al., 2021, time calibration from Russo et al., 2013, and taxonomy from Kim et al., 2021. ^1^Presence or absence of lamellocytes (Eslin and Doury, 2006; Eslin et al., 2009; Havard et al., 2009; Salazar-Jaramillo et al., 2014; Wan et al., 2019; Cinege et al., 2020). ^2^Kacsoh, 2012 documents lamellocyte-like cells from several species, sometimes at odds with reports elsewhere. Possible interpretations are discussed in the text. ^3^Presence or absence of nematocytes (Rizki, 1953; Srdic and Gloor, 1893*;* Kacsoh et al., 2014; Bozler et al., 2017). ^4^Presence or absence of multinucleated giant hemocytes (Márkus et al., 2015; Bozler et al., 2017; Cinege et al., 2020). ^5^Presence or absence of pseudopodocytes (Havard et al., 2009; Havard et al., 2012). ^6^*PPO3* is pseudogenized in *D. sechellia*; the open reading frame is interrupted by a stop codon. Kacsoh et al., 2014 found no nematocytes in *D. ananassae*, but Márkus et al., 2015 observed ‘small filariform cells.’ ^8^Unusual hemocytes reminiscent of lamellocytes were observed in infected *D. willistoni* larvae (Salazar-Jaramillo et al., 2014). ^9^Nematocytes were found in *D. willistoni* by Rizki, 1953, but not by Kacsoh et al., 2014. ^10^Kacsoh et al., 2014 mention lamellocyte homologs but no such cells were observed by Cinege et al., 2020. ^11^Bozler et al., 2017 note ‘large multicellular, and multinuclear structures.’.

Crystal cells are generally considered equivalent to the cells called oenocytoids in other insects (Lavine and Strand, 2002; Ribeiro and Brehélin, 2006; Hillyer, 2016; Eleftherianos et al., 2021), and there is plenty of evidence supporting that view. Crystal cells and oenocytoids have similar cytology, and neither cell type is known to undergo mitosis. Like crystal cells, oenocytoids are the main or sole source of phenoloxidases (Iwama and Ashida, 1986; Ashida et al., 1988), which are required for melanin deposition around parasites, at wound sites, and in pigmented cuticle. Lepidopteran oenocytoids can release their phenoloxidases in a lytic reaction, in which the cells burst and release their entire contents (Strand and Noda, 1991; Ribeiro and Brehélin, 2006). This response is triggered by prostaglandin (Shrestha and Kim, 2008; Shrestha et al., 2011; Park and Kim, 2012), and a similar prostaglandin-dependent response has also been reported from mosquitoes (Kwon et al., 2021a). Likewise, *Drosophila* crystal cells are triggered to burst at wound sites and in response to parasitization (Rizki, 1957; Rizki and Rizki, 1959; Rizki, 1978; Bidla et al., 2007; Schmid et al., 2019). One difference is that phenoloxidase is stored in regular polyhedral (‘pseudocrystalline’) inclusion bodies in the crystal cells of *D. melanogaster*, while phenoloxidases are found in the cytoplasm or in various non-crystalline inclusions in lepidopteran oenocytoids (Iwama and Ashida, 1986; Ashida et al., 1988). Mosquito oenocytoids lack cytoplasmic inclusions, and they stain homogenously for phenoloxidase in the cytoplasm (Hillyer et al., 2003). However, most drosophilids have their phenoloxidases stored in amorphous granules, as summarized in Figure 4 (Rizki and Rizki, 1980; Rizki, 1984). Crystal cells with well-ordered crystalline inclusions have only been observed in the closest relatives of *D. melanogaster*. Even within the *melanogaster* species subgroup, *D. yakuba* and *D. teissieri* have less regular inclusion bodies. Thus, there are good reasons to conclude that crystal cells are indeed oenocytoids. The term ‘crystal cell’ should either be dropped altogether (Ribeiro and Brehélin, 2006) or at least be restricted to the few species that have oenocytoids with crystalline inclusions.

#### Mosquitoes

**Figure 5.**
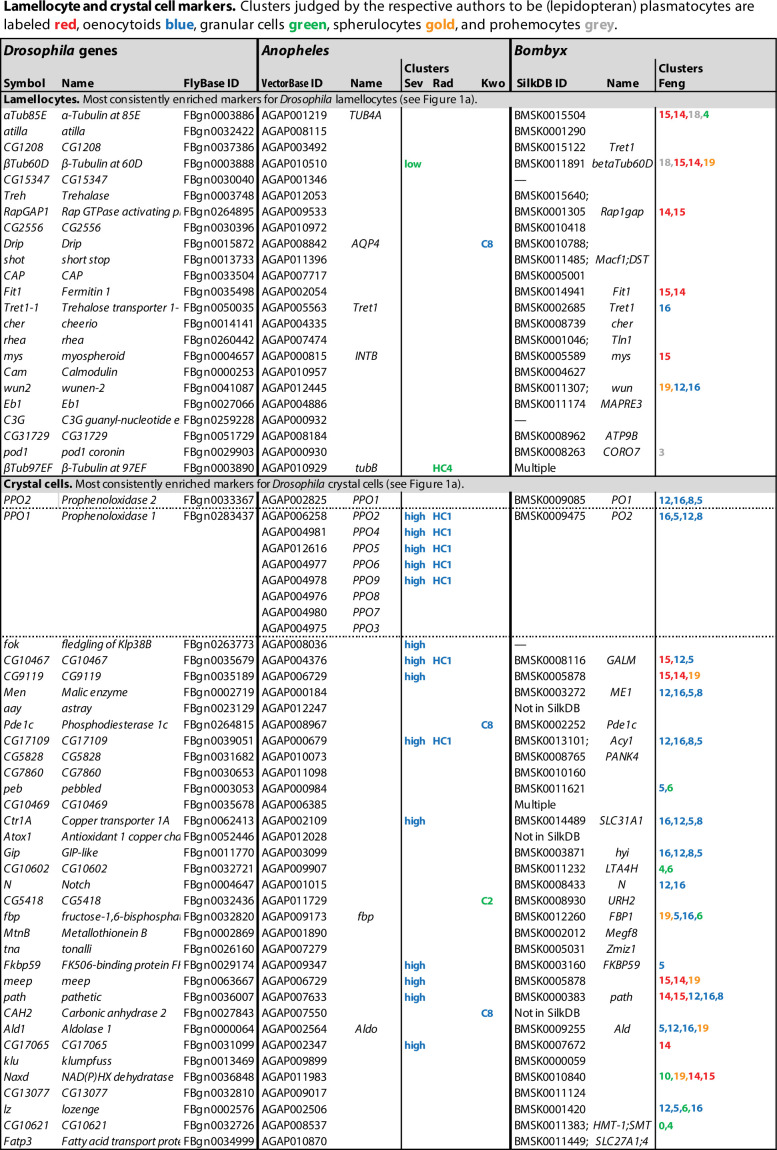
Orthologs of *Drosophila* lamellocyte and crystal cell markers expressed in mosquito and silkworm hemocyte clusters. Data from single-cell RNAseq studies by Severo et al., 2018 (Sev), Raddi et al., 2020 (Rad), Kwon et al., 2021a (Kwo), and Feng et al., 2021 (Feng). *Drosophila* markers for which no orthologs could be identified were excluded from the analysis. Clusters where the genes are significantly enriched are indicated, with highest enrichment first. Non-hemocyte clusters are omitted.

We can therefore expect that the relatedness between oenocytoids and crystal cells should also be reflected by the genes they express. Three studies on hemocytes from the malaria mosquito (*A. gambiae*) have been published, but the data do not give an entirely coherent picture (Severo et al., 2018; Raddi et al., 2020; Kwon et al., 2021b). In Figure 5, we have summarized mosquito clusters that express orthologs of *D. melanogaster* crystal cell-specific markers.

**Figure 6.**
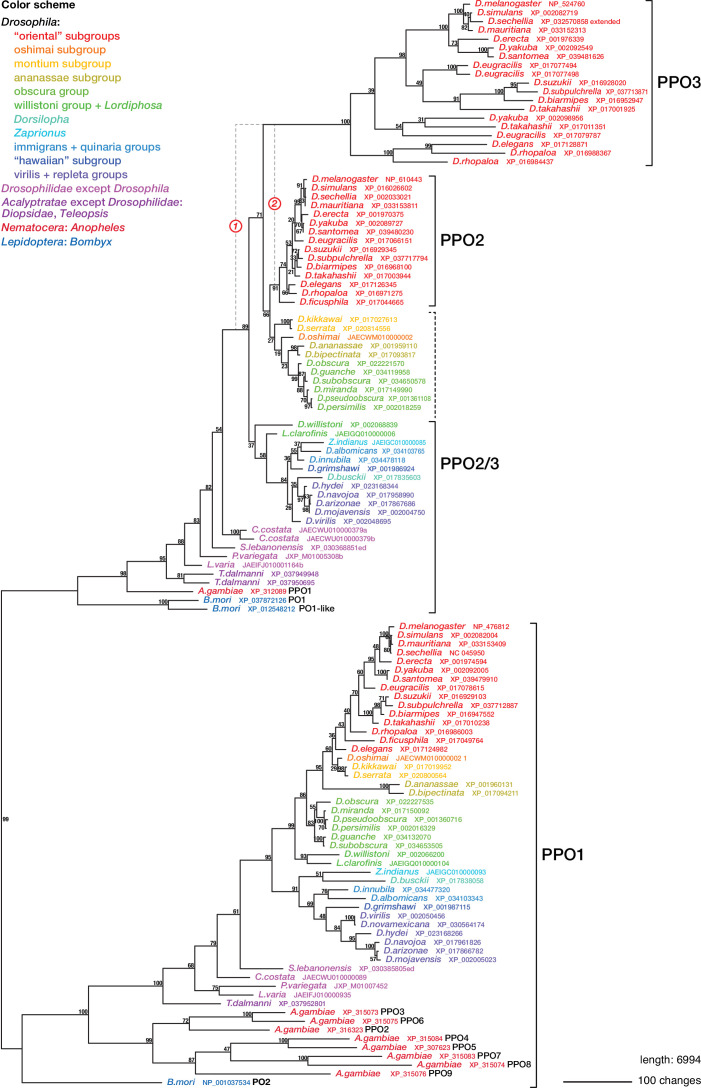
Phylogenetic relationships between insect phenoloxidases. Maximum parsimony tree of protein sequences found by blastp search of all annotated sequences from the family Drosophilidae and from *Anopheles gambiae* and *Bombyx mori*, in the refseq_protein database. Additional selected protein sequences were modeled from genomic sequences retrieved in a tblastn search of the refseq_genomes and wgs databases. Bootstrap values are percent support after 1000 replicates, using the PPO1-like proteins as outgroup. Note that the *PPO3* homolog is pseudogenized in *D. sechellia*, and there is no trace of a *PPO3* homolog in *D. ficusphila*. Consequently, although *D. sechellia* has lamellocytes, it is unable to encapsulate the eggs of parasitoid wasps (Kacsoh, 2012; Salazar-Jaramillo et al., 2014). *D. ficusphila* can encapsulate and kill parasites, but the capsules are not melanized (Kacsoh, 2012).

First, in a small study specifically focusing on oenocytoids, Severo et al., 2018 purified a population of hemocytes that express an oenocytoid-specific fluorescent marker, driven by the *prophenoloxidase 6* (*PPO6*) gene promoter. Unexpectedly, single-cell RNA sequencing of that population identified two different kinds of cells, expressing either high or low levels of the *PPO6* marker, respectively. The *PPO6* gene encodes one of the nine different phenoloxidase genes of *Anopheles*, *PPO1-PPO9. Anopheles PPO1* corresponds to the *PPO2* gene in *Drosophila*, while *Anopheles PPO2-9* are all related to *Drosophila PPO1* (Figure 6). Five of them, *PPO2*, *4*, *5*, *6,* and *9*, were more than 1000-fold enriched in the *PPO6^high^* population compared to *PPO6^low^* (Figure 5; Severo et al., 2018). The remaining four genes, *PPO1*, *3*, *7,* and *8*, were only expressed in a few scattered cells, but these cells also belonged to the *PPO6^high^* cluster. The high expression of phenoloxidase genes confirms the expectation that the *PPO6^high^* cells are oenocytoids or a subpopulation of oenocytoids. By contrast, the expression pattern of the other cluster, the *PPO6^low^* cells, corresponded to what might be expected in granular cells, with an enrichment of orthologs of *Drosophila* plasmatocyte markers as discussed below, although no morphological differences were found between *PPO6^high^* and *PPO6^low^* cells (Severo et al., 2018). The homology between the *Anopheles PPO6^high^* cells and *Drosophila* crystal cells is further supported by the fact that orthologs of several other crystal cell marker genes are enriched in the *PPO6^high^* and none in the *PPO6^low^* cluster (Figure 5). Besides the phenoloxidase genes, homologs of *CG9119*, *meep*, *Fkbp59,* and *CG17109* were all more than 1000-fold enriched in the *PPO6^high^* population (Severo et al., 2018). Homologs of *CG17065*, *Ctr1A*, *CG10467,* and *pathetic* were also enriched, but to a lesser extent. However, homologs of the classical crystal cell markers, *lozenge*, *Notch*, or *pebbled,* were not detected, perhaps due to low expression levels of these genes, and because very few cells were analyzed in this study. It should be noted that the recovery of oenocytoids was very low in this study. It may be that the lytic program of these cells was activated during the handling of the samples. In that case, the surviving cells may not be entirely representative of oenocytoids in general. It was suggested that the detection of *PPO6* marker in *PPO6^low^* cells was due to uptake of RNA-laden microvesicles, shed by the *PPO6^high^* cells (Severo et al., 2018). Alternatively, phagocytic *PPO6^low^* granular cells may have taken up fragments of disrupted oenocytoids.

**Figure 6—figure supplement 1.**
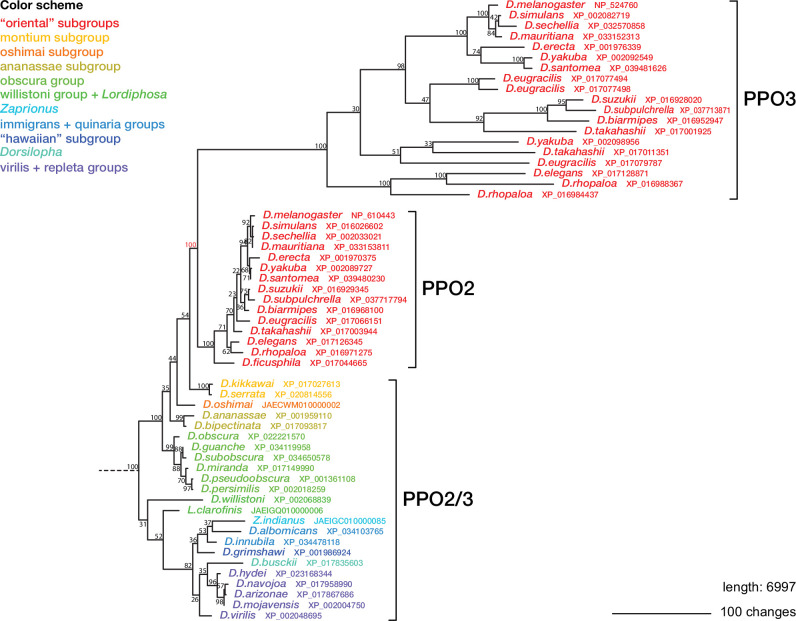
Maximum parsimony tree with forced monophyly of PPO2 + PPO3 sequences from the oriental subgroups. Data are the same as in Figure 6, but for simplicity only the *Drosophila* PPO2/3 branch of the tree is shown. Bootstrap values are percent support after 1000 replicates, using the PPO1-like proteins as outgroup.

In a larger study, Raddi et al., 2020 identified a likely oenocytoid cluster with enhanced transcription of the five phenoloxidase genes *PPO2*, *4*, *5*, *6,* and *9*. Furthermore, this cluster, called HC1, expressed two additional homologs of the *Drosophila* crystal cell markers (Figure 5), giving further support for the homology between *Drosophila* crystal cells and *Anopheles* oenocytoids. However, the enrichment of these or other transcripts was in general much lower than in the data from Severo et al., 2018. Again, none of the crystal cell markers *lozenge*, *Notch*, or *pebbled* were detected.

Unlike the other single-cell transcriptomic studies, Kwon et al., 2021b did not find statistically significant enrichment of the phenoloxidase genes in any specific hemocyte cluster. The primary markers for oenocytoids, *PPO2*, *4*, *5*, *6,* and *9*, were expressed at moderate levels, and relatively evenly distributed between seven different hemocyte clusters (Kwon et al., 2021b). One possible explanation is that the oenocytoids were completely lysed in this experiment, and that the remnants were taken up by other hemocytes. Interestingly, however, *PPO1*, *3*, *7,* and *8* transcripts were primarily found in two clusters, cluster 7 and 8, albeit at low levels. Incidentally, the latter four genes are exactly the ones that have been linked to prostaglandin-dependent induction in oenocytes of *Plasmodium*-infected mosquitoes (Kwon et al., 2021a). The same clusters were also reported to express the crystal cell markers *peb*, *DnaJ-1*, *Mlf*, *klu*, and *lozenge*, although not to levels that reached statistical significance. The authors conclude that clusters 7 and 8 correspond to the oenocytoid class, but that assignment may have to be revised, as cells in cluster 8 also express primocyte markers (see below).

#### Silkworms

A single-cell transcriptomic study of hemocytes from silkworm, *B. mori*, gives further support for a relationship between crystal cells and oenocytoids (Feng et al., 2021). In that study, no less than 20 different hemocyte clusters were identified. Four of them, numbers 5, 8, 12, and 16, were assigned to the oenocytoid class, by the criterion that they expressed the paralytic peptide-binding protein genes 1 and 2, *PPBP1* and *PPBP2*, which lack orthologs in *Drosophila* and *Anopheles*. These clusters are also highly enriched for the homologs of several crystal cell markers from *Drosophila* (Figure 5). Notably, the silkworm has three different phenoloxidase genes (Figure 6). By yet another unfortunate twist of nomenclature, the gene related to *Drosophila PPO1* is called *PO2* (or *PPO2*), and two genes related to *Drosophila PPO2* are called *PO1* and *PO1-like* (or *PPO1* and *PPO1-like*). Only PO1 and PO2 were annotated in the database used by Feng et al., and both of them were found to be highly expressed in all four oenocytoid clusters (Figure 5). Several genes involved in general metabolism and one copper ion transporter were also upregulated, presumably to meet the needs of the copper enzyme phenoloxidase. Importantly, *lozenge* transcripts were significantly enriched in all four oenocytoid clusters and *Notch* in two of them. As *lozenge* and *Notch* are characteristic markers for the crystal cell fate in *D. melanogaster* and directly involved in their hematopoiesis, this is strong evidence that crystal cells are indeed oenocytoids.

### Lamellocytes

#### Drosophilids other than *D. melanogaster*

Of particular interest are the lamellocytes, a cell type that is uniquely found only among the drosophilid flies. According to most authors, typical lamellocytes do not occur outside the genus *Drosophila*, or even outside the *melanogaster* and *suzuki* subgroups (Eslin and Doury, 2006; Eslin et al., 2009; Havard et al., 2009; Salazar-Jaramillo et al., 2014; Wan et al., 2019; Cinege et al., 2020; Figure 4). In apparent contradiction to that view, a master’s thesis by Kacsoh, 2012 documents a type of large lamellocyte-like cells in wasp-infected larvae of several other more distantly related drosophilid flies (see asterisks in Figure 4), and similar cells have also been reported from *Zaprionus indianus* and *Drosophila willistoni* (Kacsoh et al., 2014; Salazar-Jaramillo et al., 2014). They were described as large cells that flatten out on a dissection slide (Kacsoh, 2012), though not as large or flat as lamellocytes (Salazar-Jaramillo et al., 2014). It is possible that these lamellocyte-like cells correspond to the ‘activated plasmatocytes’ or to the ‘lamellocytes type II’ that have been observed in wasp-infected animals (Anderl et al., 2016; Cinege et al., 2021).

**Figure 7.**
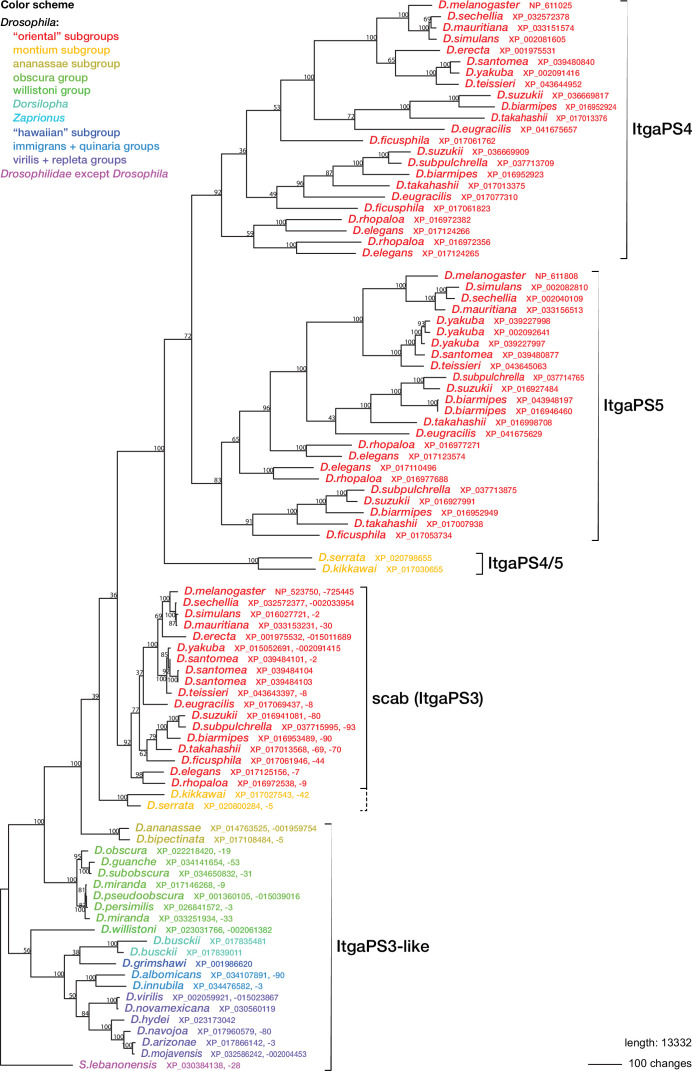
Phylogenetic relationships between *Drosophilid* integrin alphaPS3, 4, and 5 homologs. Maximum parsimony tree of protein sequences found by blastp search of all *Drosophilidae* sequences annotated in the refseq_protein database. Most ItgaPS3 and ItgaPS3-like genes have two alternative splice forms, A and B, with different approximately 63 amino acid leader sequences. The A- and B-form leaders were concatenated before the sequences were aligned. The ItgaPS4 and ItgaPS5 sequences have only an A-form leader. A few partial or chimaeric forms were excluded from the analysis. Bootstrap values are percent support after 1000 replicates, using the *Scapto Drosophila lebanonensis* protein as outgroup.

Regardless of the status of such lamellocyte-like cells, two of the more prominent lamellocyte-specific marker genes, *PPO3* and *ItgaPS4*, are uniquely present only in the genomes of the ‘oriental’ subgroups of the *melanogaster* species group (Figure 4, Figure 6, Figure 7). These are also the only species where typical lamellocytes have been found. *PPO3* and *ItgaPS4* both originate from gene duplication events in the ancestors of the ‘oriental’ species groups. It has been proposed that *PPO3* originates from a duplication of an ancestral *PPO2*-like gene (Salazar-Jaramillo et al., 2014; Dudzic et al., 2015) (node 1 in Figure 6). A more detailed phylogenetic analysis supports this idea and suggests that the duplication happened before the split between the *melanogaster* and obscura species groups (Figure 6). The exact branching order is uncertain, and a likely scenario is that the duplication actually happened even later, in the immediate ancestors of the ‘oriental’ subgroups (node 2 in Figure 6) about 20 million years ago (Figure 4). The resulting tree (Figure 6—figure supplement 1) would fit with the present distribution of the *PPO3* gene.

Similar to the *PPO3* gene, the lamellocyte marker *ItgaPS4* originates from a series of gene duplications at about time when lamellocytes first appeared on the scene. The *ItgaPS4* gene encodes an integrin alpha subunit that is expressed on the surface of lamellocytes. It is closely related to *ItgaPS5*, which is also highly enriched in hemocytes, though primarily in plasmatocytes (Leitão and Sucena, 2015). A phylogenetic analysis (Figure 7) suggests that *ItgaPS4* and *ItgaPS5* originate from the duplication of a common *ItgaPS4/5* precursor around 20 million years ago (Figure 4). Interestingly, the ancestral *ItgaPS4/5* gene in turn comes from a prior duplication of an *ItgaPS3*-like gene, maybe 10 million years earlier. Species in the *montium* subgroup still have separate *ItgaPS4/5*-like and *ItgaPS3*-like genes (Figure 4, Figure 7).

Looking at Figure 6 and Figure 7, it is striking how the evolutionary rate accelerated in the *PPO3* and *ItgaPS4* genes, immediately after they split from their rather conservative sister genes, as illustrated by their long branch lengths and complex patterns of additional gene duplications. Obviously, they must have acquired new roles, showing that the typical lamellocyte is indeed an evolutionary novelty within the ‘oriental’ lineage.

**Figure 8.**
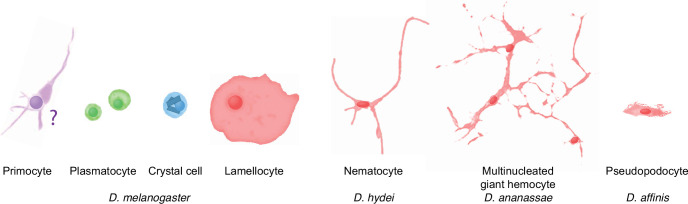
Schematic overview of drosophilid hemocyte morphologies. Plasmatocyte, crystal cell, and lamellocyte cartoons are sketched from images of *D. melanogaster* hemocytes (Rizki, 1957), the *D. hydei* nematocyte from Kacsoh et al., 2014, the *Zaprionus indianus* multinucleated giant hemocyte from Cinege et al., 2020, and the *D. affinis* pseudopodocyte from Havard et al., 2012. The primocyte illustration is based on published images of primocyte-like cells in adults (Boulet et al., 2021) and primocytes in the posterior signaling center (Krzemień et al., 2007; Mandal et al., 2007). The morphology of circulating larval primocytes is unknown.

While lamellocytes are in many ways unique, most drosophilids, like insects in general, have other specialized hemocyte types that participate in the encapsulation of parasites (Figure 8). It is an open question how these effector hemocytes are related to each other. *Nematocytes*, a characteristic class of very thin filamentous hemocytes, were first found by Rizki, 1953 in larvae of *D. willistoni*, and similar cells have later been found in *D. hydei*, *Z. indianus*, and many other drosophilids (Srdic and Gloor, 1893; Kacsoh et al., 2014; Bozler et al., 2017). Possibly related to the nematocytes are the multinucleated giant hemocytes (MGHs), first discovered in species of the *ananassae* subgroup (Márkus et al., 2015). MGHs have also been identified in *Z. indianus*, *D. falleni,* and *D. phalerata*, and perhaps *D. grimshawi* (Cinege et al., 2020; Bozler et al., 2017). The multinucleated giant hemocytes form huge and highly motile networks of fused hemocytes that ensnare parasite eggs. Together with activated plasmatocytes, they form a capsule that envelops the parasite (Cinege et al., 2021). The wide distribution of nematocytes and/or MGHs among both distant and close relatives of *D. melanogaster* (Figure 4) suggests that they must have been present already in the common ancestor of all drosophilids. Yet another type of effector cell, the pseudopodocyte, has been described from species in the *obscura* subgroup (Havard et al., 2009; Havard et al., 2012). Pseudopodocytes are large plasmatocyte-like cells equipped with numerous long pseudopods, and they participate in the encapsulation of parasites.

As the *PPO3* and *ItgaPS4* genes are markers for typical lamellocytes only, they are not informative about the possible homology between lamellocytes and other effector cells that can be found in species that lack lamellocytes. We have no single-cell transcriptomic data yet of hemocytes from drosophilids other than *D. melanogaster*, but recently the bulk transcriptome of *D. ananassae* multinuclear giant hemocytes was compared to that of other hemocytes in infected and uninfected larvae (Cinege et al., 2021). Strikingly, transcripts of one potential lamellocyte marker, the *atilla* ortholog, were found to be 300-fold enriched in the giant cells of wasp-infected larvae, compared to the circulating activated plasmatocytes in these larvae. However, this difference is mainly due to a very low expression of this gene in the latter cells. The *atilla* gene is otherwise also highly expressed in the naïve hemocytes of uninfected larvae, perhaps due to the presence of giant cell precursors. On the other hand, some lamellocyte-specific gene homologs, like the integrin *Itgbn*, are induced by infection in multinuclear giant hemocytes, and yet others, like *Trehalase*, are induced both there and in activated plasmatocytes. A substantial number of homologs of lamellocyte-specific genes are even downregulated after infection in one or both hemocyte classes. In conclusion, it is still difficult to judge if the limited overlap between gene expression in lamellocytes and multinuclear giant hemocytes is evidence of true homology or if it merely reflects an active role of these hemocytes.

#### Mosquitoes

Similarly, none of the mosquito hemocyte clusters described in the recent single-cell transcriptomic analyses (Severo et al., 2018; Raddi et al., 2020; Kwon et al., 2021b) show obvious homologies to the lamellocytes of *D. melanogaster*. Transcripts of *atilla* and CG15347 homologs are, for instance, not enriched in any hemocyte cluster (Raddi et al., 2020; Figure 5). However, we only have information from adult mosquitoes about hemocyte clustering, while lamellocytes and hemocytes with similar functions in other insects are typically found only in larvae. Thus, it is too early to speculate about possible lamellocyte homologs in mosquitoes. Besides, parasitoid wasps are certainly less of a problem for aquatic larvae.

#### Silkworms

In lepidopterans, such as *B. mori*, hemocytes called plasmatocytes (not to be confused with *Drosophila* plasmatocytes) play a similar role as the lamellocytes in *Drosophila* (Strand, 2008), and it is possible that these cell classes have a common origin in the ancestor of these insects. In line with this idea, a number of lamellocyte markers are in fact shared between *Drosophila* lamellocytes and the *Bombyx* plasmatocyte clusters 14 and 15 (Feng et al., 2021), for instance, the homologs of *α-Tubulin at 85E*, and *β-Tubulin at 60D* (Figure 5). However, the overlap is rather modest, and it could be due to convergent evolution. The *Bombyx* plasmatocyte cluster 14 also expresses mitosis markers, suggesting that unlike *Drosophila* lamellocytes these cells are mitotically active. Furthermore, a few crystal cell markers are also expressed in *Bombyx* plasmatocyte clusters 14 and 15 (Figure 5). Until we know the signaling pathways involved in their hematopoiesis, it will be difficult to judge if these cell types are related.

One additional hemocyte class has been described in lepidopterans, the *spherule cells*, which lack dipteran counterparts. Spherule cells constitute one cluster in the study of Feng et al., 2021, cluster 19. While many crystal cell orthologs are found in the silkworm oenocytoid clusters, a few are to some extent expressed in the spherule cell cluster 19 (Figure 5). These more promiscuous markers also tend to be expressed in the lepidopteran plasmatocyte clusters 14 and 15, making them less useful as indicators for a relationship to *Drosophila* lamellocytes or crystal cells.

### Primocytes

**Figure 9.**
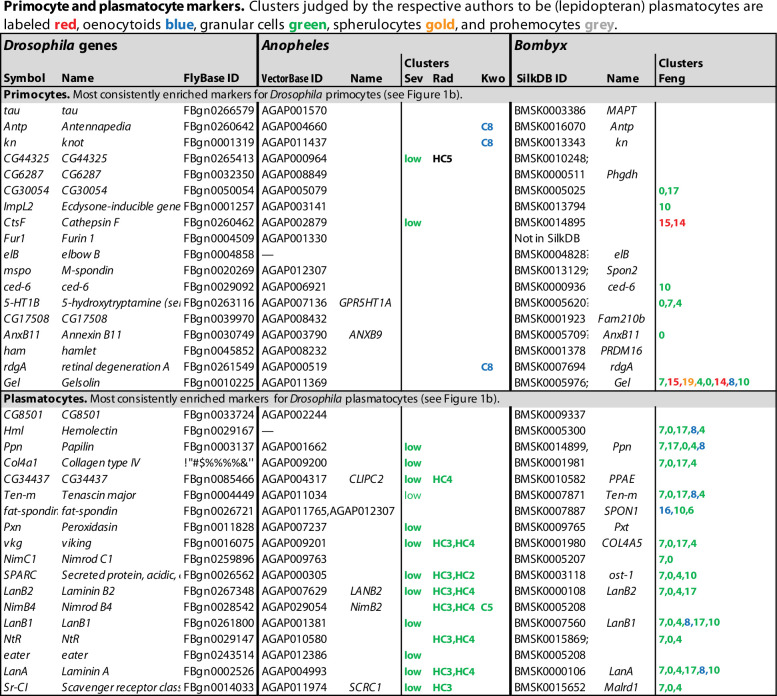
Orthologs of *Drosophila* primocyte and plasmatocyte markers expressed in mosquito and silkworm hemocyte clusters. Details as in Figure 5.

Few of the primocyte markers are enriched in any particular hemocyte cluster in mosquitoes or silkworms, and there are no convincing candidates for a primocyte class in these species. Intriguingly, as indicated in Figure 9, homologs of the key markers of primocytes, *knot* and *Antennapedia*, are enriched in the *Anopheles* hemocyte cluster 8 of Kwon et al., 2021b, but there is no indication in the other studies that these genes are expressed in any of the hemocyte classes. Cluster 8 is otherwise the candidate of Kwon et al. for being oenocytoids.

**Figure 9—figure supplement 1.**
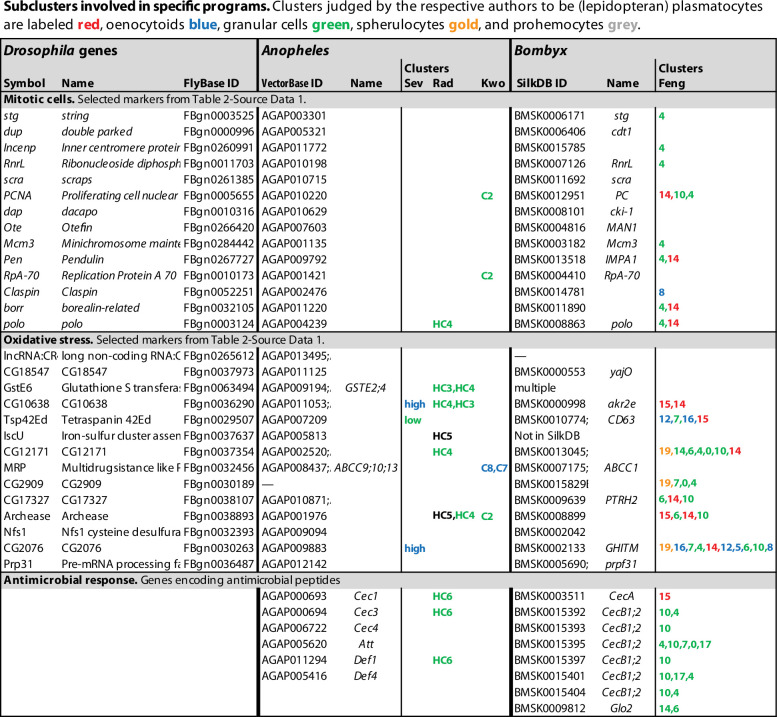
Antimicrobial peptide genes expressed in mosquito and silkworm hemocyte clusters. All antimicrobial peptide genes expressed in mosquito and silkworm hemocytes are listed. Extensive paralogies prevent us from orthology assignments. Clusters where the genes are significantly enriched are indicated, with highest enrichment first. Non-hemocyte clusters are omitted. Data come from single-cell RNAseq studies by Severo et al., 2018 (Sev), Raddi et al., 2020 (Rad), Kwon et al., 2021b (Kwo), and Feng et al., 2021 (Feng).

### Plasmatocytes/granular cells

*Drosophila* plasmatocytes have often been compared to hemocytes called granular cells in other insects since they are the major phagocytes in the respective insect groups. As a test for possible homology of these cell classes, we investigated if orthologs of our tentative *Drosophila* plasmatocyte markers were significantly enriched in particular hemocyte clusters of *Anopheles* and *Bombyx*. As shown in Figure 9, such orthologs were generally enriched in one or more of the five *Bombyx* hemocyte clusters 7, 0, 4, 17, and 10, all of which were classified as granular cells in *Bombyx* (Feng et al., 2021). The results from two of the *Anopheles* studies also support the same conclusion. Many *Drosophila* plasmatocyte markers are enriched in the granular cell-like *PPO6^low^* cluster of Severo et al., 2018, and in the main granular cell clusters HC2, HC3, and HC4 of Raddi et al., 2020. The results of Kwon et al., 2021b are less clear.

Regarding the many suggested subclusters of *Drosophila* plasmatocytes (Cattenoz et al., 2020; Tattikota et al., 2020; Fu et al., 2020; Leitão et al., 2020; Cho et al., 2020; Girard et al., 2021), most of them lack equivalents in mosquitoes or silkworms, as might be expected since they were not reproducibly found even in *Drosophila*. However, the orthologs of several markers for mitotic cells in *Drosophila* (Figure 1—source data 1) could be identified in the *Bombyx* granular cell cluster 4 (Figure 9—figure supplement 1 ), which probably includes the mitotically active fraction of granular cells in that species (Feng et al., 2021). Furthermore, as noted by Raddi et al., 2020, a minor cluster of granular cells in *Anopheles*, cluster HC6, overexpressed antimicrobial peptides, much like some minor plasmatocyte clusters in *Drosophila*. By contrast, most or all granular cell subclusters (7, 0, 4, 17, and 10) in *Bombyx* express antimicrobial peptides, but only the members of the cecropin B class, and one of the gloverins. Apparently, these peptides are constitutively expressed in silkworm granular cells, not acutely induced like in *Drosophila* and *Anopheles* (Figure 9—figure supplement 1).

## Experimental problems

Some experimental difficulties will have to be dealt with in future studies. One is the fragility of the crystal cells, which is a likely cause of the low yields of these cells in some of the studies. Crystal cells are not easy to collect, and the problem may have been exacerbated by the violent pretreatment of the larvae, intended to force the release of sessile cells. The same is true for the oenocytoids in other insects, especially for mosquitoes that have to be transfused in order to get a reasonable yield.

Other artifacts may be caused by the habit of plasmatocytes and granular cells to phagocytose fragments of other cells. Such fragments are generated when crystal cells/oenocytoids release their contents. Cell fragments may also be generated in the turnover of superfluous lamellocytes and in the autolytic disruption of larval tissue in preparation for metamorphosis. When such fragments are internalized or attached to plasmatocytes, they will contaminate the transcriptional profile of these cells. This could explain the unexpected presence of markers for crystal cells, oenocytoids, lamellocytes, or fat body cells in the plasmatocyte or granular cell transcriptomes. Further experiments will be required to resolve these issues.

The lack of reproducibility in the subclustering of *Drosophila* plasmatocytes may be due to experimental details that were not common to all laboratories. The pooling of data from parasitized and unparasitized animals may have introduced further variability. The outcome of the subclustering may also be dependent on different parameters chosen for the clustering algorithms.

The yield is a problem of its own. Most of the *Drosophila* studies were done with 15,000–20,000 hemocytes or more (Figure 1—figure supplement 1), which seems sufficient. Fu et al. assayed a smaller number, 3424, but they tested fewer conditions. Similarly, Feng et al. analyzed over 20,000 cells from the silkworm. The mosquito studies have struggled with smaller numbers. Raddi et al. assayed over 5000 cells, but Kwon et al. and Severo et al. had to do with 262 and 26 cells, respectively. This means that stochastic errors become serious, and that rare hemocyte classes will be missed. These results must therefore be regarded as tentative.

Throughout, we were surprised by the relatively modest levels of enrichment (‘FC values’) reported for many purportedly cell-type-specific transcripts. Part of the explanation may be that the borders between clusters become blurred when cells gradually activate different programs or initiate transdifferentiation, or when too many subclusters are recognized. Standard bioinformatic algorithms also tend to underestimate differences in gene expression. In order to avoid zero denominators, a constant value (typically 1) is usually added to all standardized read counts (RPKM). This gives conservative and more reliable estimates of statistical significance, but the FC values will systematically be underestimated, and the problem will become larger when the total number of reads is small.

## Conclusions and outlook

Our analysis of the recently published single-cell transcriptomic studies shows that *Drosophila* plasmatocyte heterogeneity is not due to the presence of distinct and reproducibly occurring cell classes. Rather, plasmatocytes are flexible and they have a capacity to engage in different tasks, such as production and reshaping of extracellular matrix, phagocytosis of cell debris and microbes, encapsulation of parasites, etc., (Mase et al., 2021), and to adjust their activity accordingly. The resulting heterogeneity is gradual, transient, and probably reversible, and it does not result in the formation of separate well-defined classes. A similar functional plasticity is also seen in vertebrate myeloid cells (Galli et al., 2011). In a broad sense, this capacity may be inherited from the phagocytes of early metazoans, but the more specific adaptations of these plastic cells have probably evolved independently, considering the over 600 million years of separate evolution of insects and mammals (Cunningham et al., 2017).

On the other hand, insect plasmatocytes/oenocytoids and vertebrate myeloid cells have several basal functions in common. They can phagocytize microbes and apoptotic cells, and they can detect and react to the presence of specific microbe-associated molecular patterns. These functions are probably very old, going back to the very first animals, and even to bacteria-eating protists (Gilmore and Wolenski, 2012; Franzenburg et al., 2012; Wenger et al., 2014; Menzel and Bigger, 2015; Emery et al., 2021). In *Hydra*, a jellyfish relative, these functions are carried out by phagocytic epithelial cells in the gut, while in corals like *Swiftia*, *Pocillopora,* and *Nematostella*, it is done by specialized motile immunocytes (Metchnikoff, 1892; Bosch et al., 2009; Franzenburg et al., 2012; Menzel and Bigger, 2015; Snyder et al., 2021).

In order to meet special needs of immunity and wound healing, plasmatocytes can terminally transdifferentiate to become crystal cells (oenocytoids) or lamellocytes. Oenocytoids were probably present already in the first insects, and a subclass of phenoloxidase-expressing mobile cells have even been described from the coral *Swiftia exserta* (Menzel and Bigger, 2015). By contrast, lamellocytes are products of recent and very rapid evolution. The arms race with parasites like the parasitoid wasps has brought forward a plethora of different types of highly specialized effector cells among the drosophilid flies, and typical lamellocytes can only be found in a subset of species in the genus *Drosophila*.

One novel and distinct class of hemocytes did come out of the transcriptomic studies, the primocytes. They populate the posterior signaling centers of the lymph glands, but they also appear to circulate freely in the hemolymph. It is possible that circulating or sessile primocytes play a similar role for the activation of peripheral hemocytes as the posterior signaling centers do for the cells in the lymph glands. The expression of *Antennapedia* suggests that primocytes may have an origin separate from that of other hemocytes.

A comparison of *Drosophila* crystal cell transcriptomes with oenocytoid data from *Anopheles* and *Bombyx* gives strong support for the long suspected homology of these cell types. Similarly, *Drosophila* plasmatocytes are most likely homologous to the granular cells of other insects. Unlike these well-conserved hemocyte classes, the designated effector cells of the immune defense seem to undergo very rapid evolution, generating formidable entities such as lamellocytes, multinucleated giant cells, and lepidopteran plasmatocytes.

The transcriptomic studies published so far provide a rich source of data, and further analysis can probably yield even more information. For instance, how are the changes in morphology and activity reflected in the transcriptomes of the ‘activated plasmatocytes’ in infected larvae? And, is it possible already from existing data to generate better catalogs of plasmatocyte and granular cell transcriptional markers?

## Additional files

Transparent reporting form
